# Multiple Kisspeptin Receptors in Early Osteichthyans Provide New Insights into the Evolution of This Receptor Family

**DOI:** 10.1371/journal.pone.0048931

**Published:** 2012-11-20

**Authors:** Jérémy Pasquier, Anne-Gaëlle Lafont, Shan-Ru Jeng, Marina Morini, Ron Dirks, Guido van den Thillart, Jonna Tomkiewicz, Hervé Tostivint, Ching-Fong Chang, Karine Rousseau, Sylvie Dufour

**Affiliations:** 1 Muséum National d'Histoire Naturelle, Research Unit BOREA, Biology of Aquatic Organisms and Ecosystems, CNRS 7208- IRD207- UPMC, Paris, France; 2 National Kaohsiung Marine University, Department of Aquaculture, Kaohsiung, Taiwan; 3 Leiden University, ZF-screens B.V. and Institute of Biology, Leiden, The Netherlands; 4 Technical University of Denmark, National Institute of Aquatic Resources, Charlottenlund, Denmark; 5 Muséum National d'Histoire Naturelle, UMR 7221 CNRS/MNHN Evolution des Régulations Endocriniennes, Paris, France; 6 National Taiwan Ocean University, Department of Aquaculture and Center of Excellence for Marine Bioenvironment and Biotechnology, Keelung, Taiwan; University of Rouen, France

## Abstract

Deorphanization of GPR54 receptor a decade ago led to the characterization of the kisspeptin receptor (*Kissr*) in mammals and the discovery of its major role in the brain control of reproduction. While a single gene encodes for *Kissr* in eutherian mammals including human, other vertebrates present a variable number of *Kissr* genes, from none in birds, one or two in teleosts, to three in an amphibian, xenopus. In order to get more insight into the evolution of *Kissr* gene family, we investigated the presence of *Kissr* in osteichthyans of key-phylogenetical positions: the coelacanth, a representative of early sarcopterygians, the spotted gar, a non-teleost actinopterygian, and the European eel, a member of an early group of teleosts (elopomorphs). We report the occurrence of three *Kissr* for the first time in a teleost, the eel. As measured by quantitative RT-PCR, the three eel *Kissr* were differentially expressed in the brain-pituitary-gonadal axis, and differentially regulated in experimentally matured eels, as compared to prepubertal controls. Subfunctionalisation, as shown by these differences in tissue distribution and regulation, may have represented significant evolutionary constraints for the conservation of multiple *Kissr* paralogs in this species. Furthermore, we identified four *Kissr* in both coelacanth and spotted gar genomes, providing the first evidence for the presence of four *Kissr* in vertebrates. Phylogenetic and syntenic analyses supported the existence of four *Kissr* paralogs in osteichthyans and allowed to propose a clarified nomenclature of *Kissr* (*Kissr-1* to *-4*) based on these paralogs. Syntenic analysis suggested that the four *Kissr* paralogs arose through the two rounds of whole genome duplication (1R and 2R) in early vertebrates, followed by multiple gene loss events in the actinopterygian and sarcopterygian lineages. Due to gene loss there was no impact of the teleost-specific whole genome duplication (3R) on the number of *Kissr* paralogs in current teleosts.

## Introduction

In 1999, a novel G protein-coupled receptor named GPR54 was cloned from the rat brain [1]. GPR54 ligands were later shown to be kisspeptins, previously described as metastasis suppressors, encoded by *Kiss1* gene [2]. As soon as this link between kisspeptins and GPR54 was unveiled, major discoveries in the field of reproductive endocrinology were realised. Kisspeptin and its receptor (GPR54/Kissr) emerged as major upstream regulators of the gonadotropic axis in mammals, by their key roles in the control of GnRH, mediation of steroid feedbacks, as well as initiation of puberty [3].

In mammalian species, a single gene, named *Kiss1r*, encodes for the kisspeptin receptor. To date the only exception is the platypus (*Ornithorhynchus anatinus*), a non-placental mammal, in which two receptors are present [4]. Contrasting situations are found in other tetrapods, as shown by a lack of *Kissr* in birds and up to three *Kissr* paralogous *g*enes in an amphibian species, the xenopus (*Xenopus tropicalis*) [4]. In teleosts, at least one *Kissr* is present in all species investigated so far (for review [5]). A second *Kissr* gene could be evidenced in some species including zebrafish (*Danio rerio*) [6], goldfish (*Carassius auratus*) [7], medaka (*Oryzias latipes*) [4], and striped bass (*Morone saxatilis*) [8]. However, this second paralog is lacking in the genomes of other teleosts, such as fugu (*Takifugu niphobles*), tetraodon (*Tetraodon nigroviridis*) and stickleback (*Gasterosteus aculeatus*). Concerning cyclostomes, we recently identified one *Kissr* gene in the sea lamprey (*Petromyzon marinus*) genome [9]. The homology relationships between the various *Kissr* and the evolutionary events that led to such diversity are still ambiguous.

Recently the genomes of three osteichthyan species of particular phylogenetic interest have been published: the coelacanth, *Latimeria chalumnae*, a representative of early sarcopterygians (coelacanth genome project, Broad Institute), the spotted gar, *Lepisosteus oculatus*, a non-teleost actinopterygian [10], and the European eel, *Anguilla anguilla*, a member of an early group of teleosts (elopomorphs) [11]. In the present study, we investigated the presence of *Kissr* in the genome of those relevant species.

We previously initiated the study of *Kissr* in the eel [9]. Due to its phylogenetical position, the eel may provide insights into ancestral regulatory functions in teleosts [12], the largest group of vertebrates. Furthermore, its striking biological cycle, with a blockade of sexual maturation as long as the reproductive oceanic migration is not performed, makes the eel a powerful model to investigate neuroendocrine mechanisms of puberty [13]. We formerly cloned the cDNA of one *Kissr* from the European eel brain [9].

In the present study, we report the occurrence of two additional *Kissr* in the eel, providing the first evidence for three *Kissr* in a teleost species. We also identified four *Kissr* in both coelacanth and spotted gar genomes, providing the first evidence for the presence of four *Kissr* in vertebrate species. Phylogenetic and syntenic analyses allowed us to assess the existence of four *Kissr* paralogs in osteichthyans and to raise new hypotheses on the origin and evolutionary history of vertebrate *Kissr* family. These data also let us propose a clarified *Kissr* nomenclature, based on these four paralogons. Finally, in order to get some insights into the potential process driving the conservation or the loss of multiple *Kissr*, we focused on the analyses of their tissue distributions and regulations during experimental maturation in the eel.

## Materials and Methods

### Animals

European eels (*Anguilla anguilla*) were at the prepubertal “silver” stage, which corresponds to the last continental phase of the eel life cycle, preceding the oceanic reproductive migration. Cloning and tissue distribution were performed using female and male eels purchased from Rungis International Market (Rungis, France) and transferred to MNHN, France. Eel manipulations were performed according to the guidelines of the French Ministry of Agriculture and Fisheries; Veterinary Department for Animal Health and Protection, under the supervision of authorised investigators (agreement N°I-75UPMC-F1-07). Experimental gonadal maturation was performed on farmed female eels at the DTU Aqua research facility at Lyksvad Fishfarm, Vamdrup, Denmark. Eel manipulations and experimental maturation were performed according to the guidelines of Danish Ministry of Food, Agriculture and Fisheries; Danish Veterinary and Food Administration (Approval reference for maturation experimentations: 2010/561-1783 “artificial reproduction of European eel”). Pain, suffering and stress were attempted minimized during transport and rearing throughout the experiments. All eels were anesthetized using benzocaine before sacrifice and whenever needed in relation to treatment.

### Identification of *Kissr* sequences

#### European eel genome database analysis

The TBLASTN algorithm of the CLC DNA Workbench software (CLC bio, Aarhus, Denmark) was used to retrieve the genomic sequences of three *Kissr* (named *Kissr-1*, *Kissr-2* and *Kissr-3*) from the European eel genome database [11]. The peptidic sequences of the previously characterized eel Kissr (named here Kissr-2) [9], of the two zebrafish *Kissr*, and of the three xenopus Kissr were used as queries.

#### Coelacanth and spotted gar genomic database analyses

The TBLASTN algorithm (search sensitivity: near exact matches (short)) of the *e*!ENSEMBL website was used to retrieve the genomic sequences of four coelacanth *Kissr* and four spotted gar *Kissr* from their genomic databases, respectively. The peptidic sequences of the three eel Kissr identified in the present study, of the three xenopus Kissr and of the two predicted platypus Kissr were used as queries.

The exons and splicing junctions were predicted using the empirical nucleotidic splicing signatures, *i.e.* intron begins with “GT” and ends with “AG”. The 7 transmembrane domains were determined using TMHMM software (TMHMM Server v. 2.0).

### Phylogenetic analysis

Amino-acid sequences of 51 known or predicted *Kissr* were first aligned using ClustalW [14], then manually adjusted. The JTT (Jones, Taylor and Thornton) protein substitution matrix of the resulting alignment was determined using ProTest software [15]. Phylogenetic analysis of the Kissr sequence alignment was performed using the maximum likelihood method (RaxML software [16]), with 1,000 bootstrap replicates.

Identity and similarity percentages between two sequences were calculated using EMBOSS Matcher.

### Syntenic analysis

The synteny analyses of the eel *Kissr-1*, *Kissr-2*, *Kissr-3* genomic regions were manually performed using CLC DNA Workbench 6 software and the European eel genome database. The analyses of the neighbouring genes of the four predicted spotted gar *Kissr* were performed using the preliminary gene annotation of the genome assembly LepOcu1 generated by Ensembl release 67. Synteny maps for the genomic neighbourhoods of the *Kissr* genes in human, platypus, lizard (*Anolis carolinensis*), xenopus, zebrafish, medaka, stickleback, tetraodon and coelacanth ( as well as of the corresponding region in chicken (*Gallus gallus*) were performed using the PhyloView of Genomicus v67.01 web site [17]. The analysis of the neighbouring genes of the four predicted coelacanth *Kissr* paralogs was completed by the automatic and manual gene annotation of the coelacanth genome.

### Cloning of the full-length cDNAs encoding eel Kissr-1 and Kissr-3

Total RNA from eel brain (di-/mes-encephalon) was extracted using Trizol reagent and reverse-transcripted as previously described [9]. 5′ and 3′ UTR genomic sequences of predicted *Kissr-1* and *Kissr-3* were used to design specific primers (Table S1) in order to amplify by PCR their full coding sequences (CDS). Standard PCRs were performed as follows: an initial step of polymerase activation for 3 min at 94°C; then 40 cycles with 30 s at 94°C for denaturing, 30 s at various temperatures (54°C for *Kissr-1* and 66°C for *Kissr-3*) for annealing, 1 min 30 s at 72°C for primer extension, and a single final extension step of 5 min at 72°C. PCR products were purified with the QUIAquick PCR Purification Kit (Qiagen, Hilden, Germany) and inserted in a pCR™4-TOPO® TA vector provided by the TOPO® TA Cloning® Kit (Invitrogen). The vectors were then transfected in One Shot® TOP10 Chemically Competent *E. coli* (Invitrogen). After the bacteria containing a vector with insert had grown in miniprep cultures, vectors were extracted and purified using QUIAquick Spin Miniprep Kit (Qiagen, Hilden, Germany). Their inserts were then sequenced at GATC biotech Ltd (Konstanz, Germany). The obtained sequences were submitted to EMBL.

### *Kissr* tissue distribution in the eel

Various tissues were collected from eight freshwater female silver European eels to investigate the distribution of *Kissr-1*, *Kissr-2* and *Kissr-3* expressions, using qPCR. Eels were sacrificed by decapitation. The following organs were sampled, stored in RNAlater (Ambion-Inc, Austin, TX, USA) and kept frozen at −20°C until RNA extraction: brain, pituitary, ovary, muscle, eye, liver, adipose tissue, kidney, intestine and spleen. The brain was dissected into five parts [18]: olfactory bulbs, telencephalon, di-/mes-encephalon, *cerebellum* and *medulla oblongata*. In addition, testes from eight freshwater male silver European eels were also sampled.

### Eel experimental maturation

Farmed female silver European eels transferred to seawater (36‰) received weekly injections of salmon pituitary extract for four months to induce vitellogenesis followed by one dihydroxy-progesterone injection to induce final oocyte maturation and ovulation, according to [19]. Analyzes were performed on nine matured eels and seven controls. After sacrifice, anterior brain divided in two parts (olfactory bulbs/telencephalon; di-/mesencephalon), pituitary and ovary were dissected and stored in RNAlater and kept frozen at −20°C until RNA extraction.

### Quantitative real-time PCR (qPCR)

Eel *Kissr-1*, *Kissr-2* and *Kissr-3* specific primers (Table S1) were designed based on the full-length European eel CDS sequences cloned in this study (*Kissr-1* and *Kissr-3*) and previously [9] (*Kissr-2*), using Primer3 Software (Whitehead Institute/Massachusetts Institute of Technology, Boston, MA). The *Kissr-3* qPCR primers were designed in exon-3, to quantify the expression level of the three *Kissr-3* mRNA splicing isoforms at once. To optimize the assays, different annealing temperatures were tested according to the melting temperature (Tm) of primers. To assess their specificity, amplification products were sequenced at GATC Biotech Ltd. Primers for European eel *LHβ*, *mGnRH* and reference gene *β-actin* were as previously designed [18], [20] (Table S1). All primers were purchased from Eurofins (Ebersberg, Germany).

Quantitative assays of eel *Kissr-1*, *Kissr-2*, *Kissr-3*, *mGnRH*, *LH-β* and *β-actin* mRNAs were performed using the LightCycler® System (Roche, Ltd. Basel, Switzerland) with SYBER Green I sequence-unspecific detection as previously described [18], [20], [21]. The qPCR primers are listed in Table S1. The qPCRs were prepared with 4 µl of diluted cDNA template, 2 µl of PCR grade water, 2 µl of SYBR Green master mix and 1 µl of each forward and reverse primer (0.5 pmole each at final concentration). The qPCRs were performed as follows: an initial step of polymerase activation for 10 min at 95°C; then 41 cycles with 15 s at 95°C for denaturing, 5 s at 60°C (*Kissr-1*, *Kissr-2*, *mGnRH*, *LH-β* and *β-actin*) or 62°C (*Kissr-3*) for annealing, 10 s at 72°C for primer extension, 5 s at 83°C to avoid measurement of non-specific annealing; and a single final extension step of 5 min at 72°C. Each qPCR run contained a non-template control (cDNA was substituted by water) for each primer pairs to confirm that reagents were not contaminated. The efficiency of all primers was tested and the specificity of each reaction was assessed by melting curve analysis to ensure the presence of only one product, and by sequencing. Individual tissue samples were then analyzed in duplicate by qPCR. Serial dilutions of cDNA pool of brain tissues were run in duplicate and used as a common standard curve and also included in each run as a calibrator. Normalisation of data was performed using total RNA content for the tissue distribution samples, and using *β-actin* mRNA level for experimental maturation samples.

### Statistical analysis

Results are given as mean ± SEM. Means were compared by Student's *t*-test using Instat (GraphPad Software Inc., San Diego, Calif., USA).

## Results and Discussion

### Characterization of three European eel *Kissr*

#### Genomic prediction of three eel *Kissr* genes

We used the deduced peptidic sequence from the previously cloned European eel *Kissr* cDNA [9] (considered as *Kissr-2* in the present study) as a query to retrieve *Kissr* sequences in the European eel genome. TBLASTN results revealed the presence of three different *Kissr* genes, called *Kissr-1*, -2, -3, each made of 5 exons and 4 introns (Fig. S1), that constitutes the conserved structure of *Kissr* genes. The lengths of exon-2, exon-3 and exon-4 are the same for all three genes with 125 bp, 136 bp and 239 bp, respectively. The exon-1 of *Kissr-1*, *Kissr-2* and *Kissr-3* is 235 bp, 214 bp and 229 bp, respectively. The exon-5 is 348 bp, 372 bp and 351 bp, respectively. This leads to predicted *Kissr-1*, *Kissr-2* and *Kissr-3* CDS of 1083 bp, 1086 bp and 1080 bp, respectively.

#### Cloning of eel *Kissr* cDNAs

**Figure 1:**
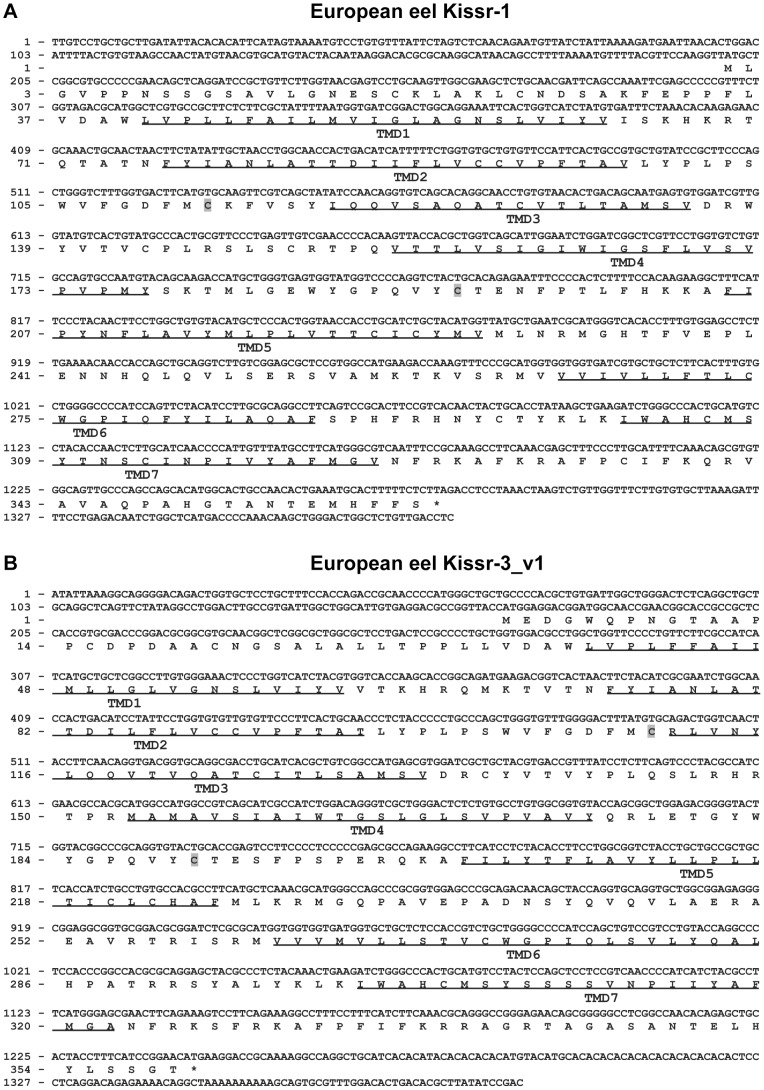
Molecular cloning of eel *Kissr-1* and *Kissr-3_v1*. Nucleotide and deduced amino-acid sequence of the cDNA encoding the eel *Kissr-1* (A) and *Kissr-3_v1* (B). Nucleotides (top) are numbered from 5′ to 3′. The amino-acid residues (bottom) are numbered beginning with the first methionine residue in the ORF. The asterisk (*) indicates the stop codon. The predicted seven transmembrane domains (TMD) are underlined and the cysteines involved in a disulphide bridge are shaded in grey.

Using European eel specific *Kissr-1* primers, PCRs performed on brain cDNAs led to a single product. Its sequence (EMBL: HE802271) encompassed partial 5′ and 3′ UTRs of 199 bp and 102 bp, respectively, and a full CDS of 1083 bp. Once translated, the cloned *Kissr-1* CDS gives a 360-aa receptor, exhibiting the seven transmembrane domains (TMD) characteristic of the GPCR family (Fig. 1A).

The previously cloned eel *Kissr* cDNA [9] is named *Kissr-2* in the present study.

Using European eel specific *Kissr-3* primers, PCRs performed on brain cDNAs led to the isolation of three different products (EMBL: HE802272, HE802273 and HE802274). Their sequence revealed three *Kissr-3* isoforms – named here *Kissr-3*_v1 (Fig. 1B), *Kissr-3*_v2 and *Kissr-3*_v3 (Fig. S2). The partial 5′ and 3′ UTR are 167 and 147 bp for all three isoforms. The *Kissr-3*_v1 isoform corresponds to the 5 predicted exons from the eel genome. Once translated, it gives a 359-aa receptor exhibiting the seven conserved TMD (Fig. 1B). The *Kissr-3*_v2 CDS corresponds to the 5 predicted exons, minus the 76 first nucleotides of the exon-2 that are missing (Fig. S2A). The loss of these 76 nucleotides results in a shift of the *Kissr-3* reading frame and the occurrence of a first premature termination codon at position 280 pb. Once translated, it gives a 93-aa protein. This shorter protein exhibits a single TMD encoded by exon-1. The *Kissr-3*_v3 CDS corresponds to exon-1, exon-3, exon-4 and exon-5, while exon-2 is completely missing (Fig. S2B). The loss of exon-2 results in a shift of the *Kissr*-3 reading frame without the occurrence of any premature termination codon. Once translated, it gives a 361-aa protein, which exhibits only one TMD encoded by exon-1. The presence of the three *Kissr-1*, *-2*, and *-3* transcripts and of the three *Kissr-3* mRNA isoforms was also observed in Japanese eel (*Anguilla japonica*) brain cDNAs (data not shown).

To date, only one or two *Kissr* genes had been found in the genome of teleosts (reviewed by [5]), so the eels provide the first evidence of the presence of three *Kissr* genes in teleosts. Until now three *Kissr* genes had been found only in an amphibian species, xenopus [4].

In addition to this *Kissr* gene diversity, European and Japanese eels present three *Kissr-3* mRNA isoforms revealing similar alternative splicing in both species. The existence of *Kissr* mRNA splicing isoforms has been described in two other teleosts, the sole (*Solea senegalensis*) [22] and the zebrafish [23]. In the sole, two isoforms were identified for the single *Kissr* (*Kissr-2* type) gene present in this species and they differ by the retention of intron III, while in the eel they differ by partial or complete deletion of exon-2 in *Kissr-3* [22]. In the zebrafish, five isoforms were identified for *Kiss1rb* (*Kissr-3* type), while no splicing isoform was observed concerning *Kiss1ra* (*Kissr-2* type). Among the five zebrafish *Kiss1rb* isoforms, one of them, called *KRBDP1*, resulted from the deletion of exon-2 and corresponds to eel *Kissr-3_v3* [23]. Even though the existence of other *Kissr* mRNA isoforms in the eel cannot be excluded, the three genes and several isoforms described here highlight the *Kissr* molecular diversity in basal teleosts. They also could reflect an ancestral post-transcriptional regulatory process of *Kissr*.

### Genomic prediction of four coelacanth and four spotted gar *Kissr* genes

To further assess the *Kissr* diversity in vertebrates, we investigated the presence of these genes in the genomes of the spotted gar, a non-teleost actinopterygian, and the coelacanth, a basal sarcopterygian, two species of relevant phylogenetical positions. We performed a TBLASTN in both genomes (Ensembl) using the three European eel, the two zebrafish and the three xenopus Kissr proteins as queries. The TBLASTN results revealed the existence of four *Kissr* genes in both coelacanth and spotted gar genomes, named here *Kissr-1*, *Kissr-2*, *Kissr-3* and *Kissr-4*.

#### Four *Kissr* genes in the coelacanth genome

The four predicted *Kissr* genes of coelacanth are made of 5 exons and 4 introns. The predicted CDS and the exon-exon junctions of each putative transcript are shown in Fig. S3. Once translated, the putative transcripts lead to four predicted proteins, *i.e.* coelacanth Kissr-1, Kissr-2, Kissr-3 and Kissr-4, of 368-aa, 367-aa, 377-aa and 367-aa, respectively (Fig. S3).

#### Four *Kissr* genes in the spotted gar genome

The four predicted *Kissr* genes of spotted gar are also made of 5 exons and 4 introns. Although *Kissr-1* exon-4 and *Kissr-4* exon-5 are partial due to the current status of the genome, the predicted CDS and the exon-exon junctions of each putative transcript are shown in Fig. S4. Once translated, the putative transcripts lead to four predicted proteins, *i.e.* spotted gar Kissr-1 to 4 of 318-aa, 362-aa, 382-aa, and 385-aa, respectively (Fig. S4).

In both coelacanth and spotted gar, all predicted Kissr proteins present the typical seven TMD of the GPCR family (Fig. S3 and S4).

These new findings evidence for the first time the potential existence of four *Kissr* paralogs in vertebrate species, representatives of the sarcopterygian (coelacanth) and the actinopterygian (spotted gar) lineages.

### Phylogenetic analysis of the *Kissr* family

#### Other genomic database analyses

In addition to the characterisation and/or the prediction of European eel, coelacanth and spotted gar *Kissr*, we used the ENSEMBL Genome Browser to retrieve the sequences of two *Kissr* in the medaka genome (GPR54-1, chromosome 17 and GPR54-2, chromosome 9; [4]), and to search for new candidate *Kissr* genes in the genomes of other vertebrates. We found one putative *Kissr* gene, named here *Kissr-2*, in several teleost genomes, stickleback (chromosome group III), tetraodon (chromosome 15-random) and cod (*Gadus morhua*; scaffold 377), one *Kissr* in a cyclostome genome, sea lamprey (scaffold GL478157; [9]), and one in the lizard genome (chromosome 2).

#### Phylogenetic analysis

**Figure 2:**
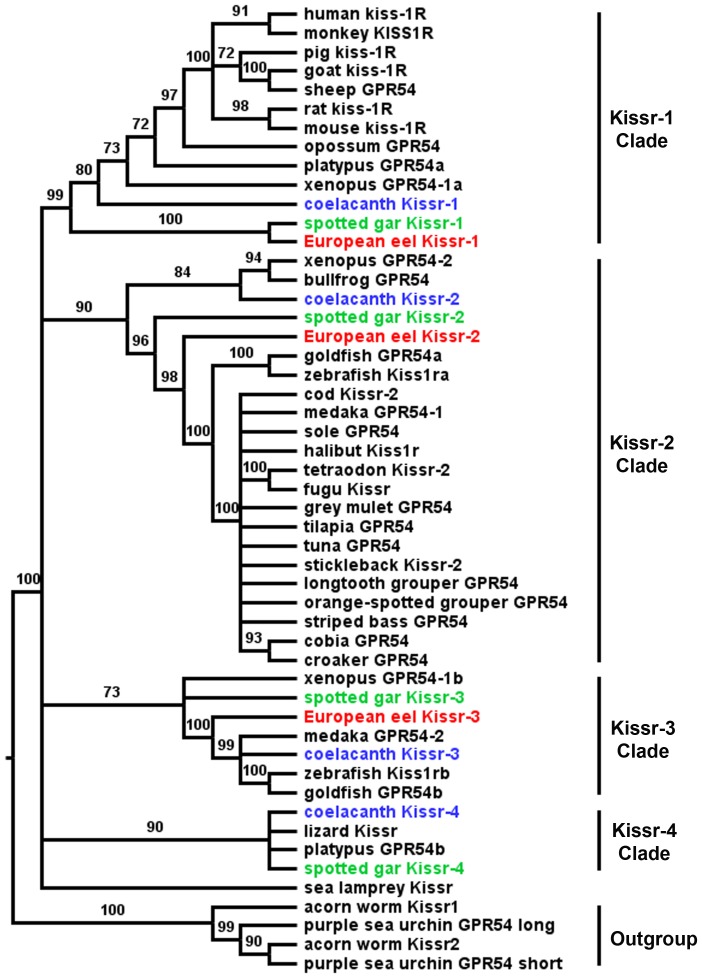
Consensus phylogenetic tree of the vertebrate kisspeptin receptors (Kissr). This phylogenetic tree was constructed based on the amino-acid sequences of Kissr (for the references of each sequence see Table S2) using the Maximum Likelihood method with 1,000 bootstrap replicates. The number shown at each branch node indicates the bootstrap value (%); only values and branching above 70% are indicated. The tree was rooted using the two sequences of the hemichordata acorn worm GPR54-1 and GPR54-2 and the two sequences of echinodermata purple sea urchin Kissr_short and Kissr_long. The European eel Kissr and predicted coelacanth and spotted gar Kissr are coloured in red, blue and green, respectively.

Based on an alignment of 51 Kissr peptidic sequences (Fig. S5), and assuming ambulacrarian (acorn worm, *Saccoglossus kowalevskii*, and purple sea urchin, *Strongylocentrotus purpuratus* (Table S2)) Kissr as outgroup, a phylogenetic tree was generated using the Maximum Likelihood method (the list of sequences and accession numbers is provided in Table S2). As shown in Fig. 2, it clusteres the vertebrate Kissr into four main clades, which are supported by significant bootstrap values (99, 90, 73, and 90%, respectively). Based on this analysis and the determination of those four clades, we propose the following new nomenclature of the different Kissr.

The first clade mainly encompasses sarcopterygian Kissr including all eutherian Kissr, a metatherian Kissr (*Monodelphis domestica*; opossum GPR54), a prototherian Kissr (platypus GPR54a), a xenopus Kissr (GPR54-1a) and coelacanth Kissr-1. In addition, two actinopterygian Kissr, spotted gar and European eel Kissr-1, branch together at the base of this clade. This is the first evidence of the presence of an ortholog to eutherian Kissr in actinopterygians. Acknowledging the presence of eutherian (including human) Kissr, which have been the first to be discovered, this clade was named Kissr-1 clade. The eel is up to now the only teleost presenting an ortholog (eel Kissr-1) to mammalian Kissr.

The second clade, named Kissr-2, clusters mainly actinopterygian Kissr, *i.e.* spotted gar Kissr-2 and most of the previously described teleost Kissr including zebrafish Kiss1ra, goldfish GPR54a, medaka GPR54-1 and European eel Kissr-2. This clade also clusters together three sequences from sarcopterygian species, xenopus GPR54-2, bullfrog (*Rana catesbeiana*) GPR54 and coelacanth Kissr-2.

The third clade, named Kissr-3, clusters two sarcopterygian Kissr (xenopus GPR54-1b and coelacanth Kissr-3) and some actinopterygian Kissr (spotted gar Kissr-3 and a few teleost Kissr: zebrafish Kiss1rb, goldfish GPR54b, medaka GPR54-2 and European eel Kissr-3).

The fourth clade, named Kissr-4, clusters three sarcopterygian Kissr (platypus GPR54b, lizard Kissr and coelacanth Kissr-4) with one actinopterygian Kissr, the spotted gar Kissr-4.

This phylogenetic analysis suggests the existence of four distinct paralogous Kissr in osteichthyans. Furthermore, it shows that each sarcopterygian Kissr is orthologous to actinopterygian Kissr. This is specially highlighted by the relationship between the four coelacanth and spotted gar Kissr.

### Syntenic analysis of *Kissr* genes

**Figure 3:**
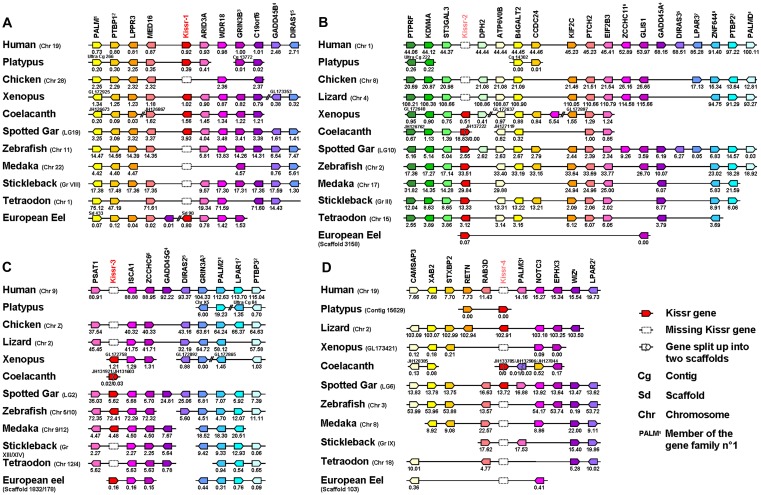
Conserved genomic synteny of osteichthyan *Kissr*. Genomic synteny maps comparing the orthologs of *Kissr-1* (A), *Kissr-2* (B), *Kissr-3* (C), *Kissr-4* (D) loci and their neighbouring genes. Analyses were performed on the genomes of human (*Homo sapiens*), platypus (*Ornithorhynchus anatinus*), lizard (*Anolis carolinensis*), chicken (*Gallus gallus*), xenopus (*Xenopus (Silurana) tropicalis*), coelacanth (*Latimeria chalumnae*), spotted gar (*Lepisosteus oculatus*), zebrafish (*Danio rerio*), medaka (*Oryzias latipes*), stickleback (*Gasterosteus aculeatus*), tetraodon (*Tetraodon nigroviridis*) and European eel (*Anguilla anguilla*). This map was established using the PhyloView of Genomicus v67.01 web site, manual annotation of European eel genome using CLC DNA Workbench 6 software and the gene annotation of the coelacanth and spotted gar genomic databases (see section 2). *Kissr* genes are named according to our proposed nomenclature (*Kissr-1* to *Kissr-4*). The other genes are named after their human orthologs according to Human Genome Naming Consortium (HGNC). Orthologs of each gene are shown in the same color. Each of the eight conserved gene families is identified by an exponent number. The direction of arrows indicates the gene orientation, with the position of the gene (in 10^−6^ base pairs) indicated below. The full gene names and detailed genomic locations are given in Table S3.

In order to test the results obtained with the phylogenetic analysis, we performed a syntenic analysis of the *Kissr* neighbouring genes, an approach which is applied to determine gene orthology relationships as well as the origin and evolutionary history of gene families. For this analysis (Fig. 3), we considered the following vertebrate representatives: mammals (eutherian: human and prototherian: platypus), bird (chicken), squamate (lizard), amphibian (xenopus), basal sarcopterygian (coelacanth), non-teleost actinopterygian (spotted gar) and teleosts (zebrafish, medaka, stickleback, tetraodon and European eel). As already reported (for review: [5], [24]–[26]), genomic synteny analysis shows that birds do not possess any *Kissr* gene.

The mammalian, amphibian, coelacanth, spotted gar and European eel genes from Kissr-1 clade are positioned in genomic regions containing common loci, including *PALM*, *PTBP1*, *LPPR3*, *MED16*, *ARID3A*, *WDR18*, *GRIN3B*, *C19orf6*, *GADD45B* and *DIRAS1*, exhibiting well conserved synteny (Fig. 3A). This supports the orthology of the *Kissr-1* genes. Syntenic analysis suggests that the other teleost genomes do not contain any *Kissr-1* gene, though the above-mentioned neighbouring genes are present in the corresponding genomic regions (Fig. 3A). The eel currently provides a unique example of *Kissr-1* ortholog in teleosts.

The amphibian, coelacanth, spotted gar and teleost genes from Kissr-2 clade are positioned in genomic regions containing common loci, including *PTPRF*, *KDM4A*, *ST3GAL3*, *DPH2*, *ATPV60B*, *B4GALT2*, *CCDC24*, *KIF2C*, *PTCH2*, *EIF2B3*, *ZCCHC11*, *GLIS1*, *GADD45A*, *DIRAS3*, *LPAR3*, *ZNF644*, *PTBP2* and *PALMD*, exhibiting well conserved synteny (Fig. 3B). This supports the orthology of the *Kissr-2* genes. European eel *Kissr-2* orthology is only supported by the presence of partial *GLIS1* due to the small size of the scaffold. Syntenic analysis suggests that squamate (lizard) and mammalian genomes do not contain any *Kissr-2* gene, though the above-mentioned neighbouring genes are present in the corresponding genome regions (Fig. 3B).

The amphibian, teleost (zebrafish, medaka, European eel) and spotted gar *Kissr-3* genes are positioned in genomic regions containing common loci including *PSAT1*, *ISCA1*, *ZCCHC6*, *GADD45G*, *DIRAS2*, *PALM2*, *LPAR1* and *PTBP3*, exhibiting conserved synteny (Fig. 3C). This supports the orthology of the *Kissr-3* genes. Syntenic analysis suggests that the other teleost and tetrapod genomes do not contain any *Kissr-3* gene, though the above-mentioned neighbouring genes are present in the corresponding genome regions. The coelacanth predicted *Kissr-3* is split into the scaffolds JH131603.1 and JH131921.1, which are too small to contain any other gene (Fig. 3C).

Platypus and lizard *Kissr-4* gene neighbouring regions present only *RETN* gene in common, due to the small size of the platypus scaffold (Fig. 3D). Assemblage of three scaffolds (JH133705.1, JH132986.1, and JH127844) from coelacanth genome allowed us to reveal a *Kissr-4* neighbouring region comprising *LPAR2*, *STXBP2* and *EPHX3* genes, with *STXBP2* and *EPHX3*, being also present in lizard and spotted gar *Kissr-4* region (Fig. 3D). These data suggest that *Kissr-4* genes can be considered as orthologous. Syntenic analysis also suggests that the genomes from placental mammals, amphibian and teleosts do not contain any *Kissr-4* gene, though they present in a common region some conserved genes including *CAMSAP3*, *XAB2*, *STXBP2*, *RETN*, *RAB3D*, *PALM3*, *NOTCH3*, *EPHX3*, *WIZ* and *LPAR2* (Fig. 3D).

This syntenic analysis of *Kissr* genes delineated four different conserved genomic regions among osteichthyans. For each conserved genomic region, *Kissr* genes from various species clustered in the corresponding phylogenetic Kissr clade. Thus, the results of the syntenic analysis fully validated the orthology relationships of the phylogenetic analysis and further supported our proposal for a new nomenclature.

### Evolutionary history of *Kissr* family

#### Origin of the four *Kissr* present in basal osteichthyans

So far, the presence of *Kissr* has been characterised in two non-vertebrate species, the purple sea urchin and the acorn worm (Table S2). This revealed the existence of at least one ancestral *Kissr* before the vertebrate emergence. The predictions of four *Kissr* in the genomes of a basal sarcopterygian, the coelacanth, and of an actinopterygian, the spotted gar, together with the results of both phylogenetic and syntenic analyses, enabled us to hypothesise the existence of at least four *Kissr* paralogous genes in the common osteichthyan ancestor of the sarcopterygian and actinopterygian lineages. A remaining question was to infer whether these four genes resulted from the duplications of one or multiple ancestral *Kissr* genes.

It is currently admitted that two rounds of whole genome duplication (1R and 2R) occurred in the early vertebrate evolutionary history, resulting in four-fold replicates of the ancestral genome. Even though numerous genomic rearrangements and loss events occurred during the vertebrate radiation, the vestiges of these two successive genome duplications can be revealed in the current vertebrate species by the existence of numerous four-fold repeated regions (tetra-paralogon) carrying paralogous genes [27].

**Figure 4:**
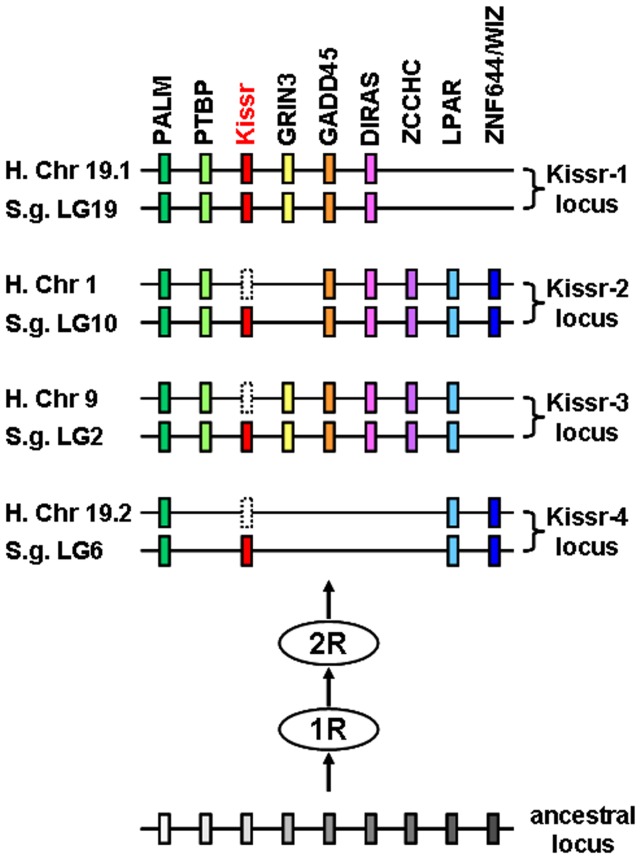
Proposed origin of osteichthyans *Kissr* loci based on human and spotted gar *Kissr* tetra-paralogons. The paralogous genes of each of the eight identified families delineate four tetra-paralogons in both human and spotted gar. This suggests a common origin of the four loci before the two whole genome duplication rounds (1R and 2R) occurred in the early vertebrate history.

Our syntenic analysis demonstrated that the current osteichthyan *Kissr* genes are localised in four different genomic regions. In the Fig. 3, in addition to the *Kissr* genes, we could reveal paralogs from eight gene families present among the four syntenic regions of the osteichthyan representative species. The eight considered families are PALM (4 paralogs), PTBP (3 paralogs), GRIN3 (2 paralogs), GADD45 (3 paralogs), DIRAS (3 paralogs), ZCCHC (2 paralogs), LPAR (3 paralogs) and ZNF644/WIZ (2 paralogs). Fig. 4 focuses on the comparison of these four conserved *Kissr* syntenic regions in one sarcopterygian, the human, and one actinopterygian, the spotted gar, two species chosen for their genomic assembly in chromosomes and their phylogenetic positions among osteichthyans. Fig. 4 shows that the members of these eight gene families delineate a tetra-paralogon with the four conserved genomic regions of both human and spotted gar. This syntenic observation strongly suggests that the four *Kissr* resulted from “en-bloc” duplications of a unique ancestral genomic region.

**Figure 5:**
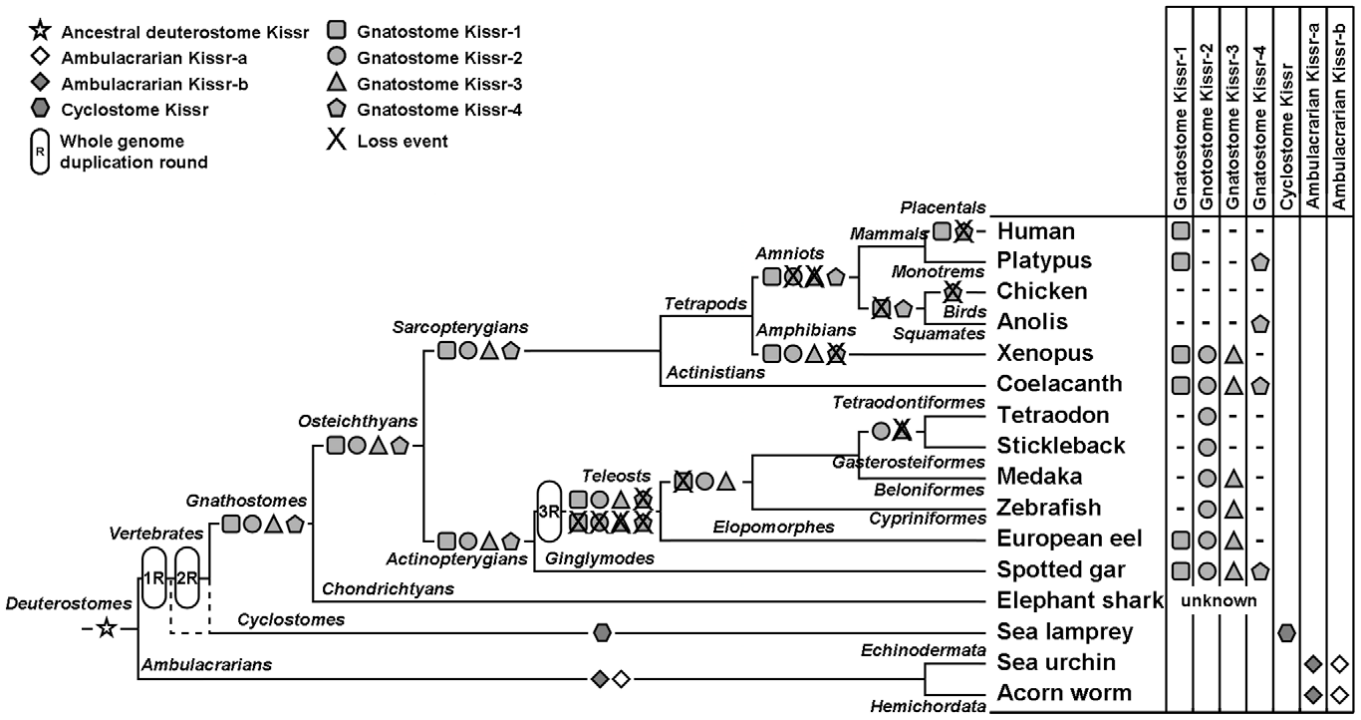
Current status and proposed evolutionary history of *Kissr* family. This representation is based on the phylogenetic and synteny analyses. The names of the main phyla are given on the corresponding branches. The names of the current representative species of each phylum are given at the end of the final branches, together with the symbol of the *Kissr* gene they possess. This hypothesis assumes the presence of the four *Kissr* paralogs in the osteichthyan lineage resulting from the two rounds of vertebrate whole genome duplication. Multiple subsequent *Kissr* gene loss events are indicated in the actinopterygian and sarcopterygian lineages.

Recently, two studies proposed reconstructions of the genomes of vertebrate and chordate ancestors. In the first study, ten proto-chromosomes of the ancestral vertebrate karyotype and their linkage to the corresponding tetra-paralogons in the human genome were hypothesised [28]. The second study proposed a reconstruction into seventeen proto-chromosomes of the last chordate ancestor genome and their linkage to the human tetra-paralogons [29]. Considering these two studies, together with our localisation of the four *Kissr* syntenic regions in the human genome, we can hypothesise that the corresponding tetra-paralogons resulted from the duplications of one single region localised on the proto-chromosome-A of the vertebrate ancestor and on the proto-chromosome-1 of the last chordate ancestor. From these analyses, we can infer that the four *Kissr* paralogons may have resulted from the two successive rounds of whole genome duplication (1R and 2R) that occurred in basal vertebrates (Fig. 5).

To date, the exact timing of the 2R occurrence is uncertain and the impact of this event on the cyclostomes is still debated [30], [31]. In our study, neither phylogenetic nor syntenic analyses enabled us to specifically relate the sea lamprey *Kissr* to one of the four osteichthyan *Kissr*. The currently available data also led to a polytomy of the four Kissr clades and did not cluster them into two major clades, each of them divided in two sub-clades, which would have reflected the successive 1R and 2R. In the future, increasing number of characterised Kissr sequences, especially from Kissr-3 and Kissr-4 clades, may bring sufficient new information to resolve this polytomy. Though kisspeptin genes have been characterised in chondrichthyans, it could be of particular interest to investigate the presence of its receptor in this lineage to further assess *Kissr* history in early gnathostomes.

#### A subsequent history of losses

Our study suggests that four *Kissr* paralogs would have been present in ancestral gnathostomes, resulting from 1R and 2R. It also shows that these four paralogons are still present in two early emerged osteichthyans, a sarcopterygian, the coelacanth and an actinopterygian, the spotted gar. All other vertebrate species investigated so far possess less *Kissr* genes (from 3 to none) indicating multiple events of *Kissr* losses in both the sarcopterygian and actinopterygian lineages (Fig. 5).

In tetrapods (sarcopterygians), *Kissr-4* would have been lost in amphibians, *Kissr-1*, *-2* and *-3* being present in the xenopus, while in amniotes the losses would have first concerned *Kissr-2* and *Kissr-3*, *Kissr-1* and *Kissr-4* being present in a prototherian mammal (Fig. 5). Further alternative losses occurred in amniotes, with only *Kissr-1* remaining in eutherian mammals, but only *Kissr-4* in squamates (lizard) (Fig 5). Finally, an additional loss would have led to the complete absence of *Kissr* in birds.

In teleosts (actinopterygians), a third round of whole genome duplication (3R) is supposed to have occurred specifically in the early history of this group. The 3R is usually considered as one of the main factors that drove the large radiation and adaptative success of the teleost lineage [11], [32]. As four *kissr* were present in basal actinopterygians, the teleost-specific 3R implied the potential existence of up to eight *Kissr* genes in the early teleost history. However, our results show that the largest number of *Kissr* exhibited by current teleosts is three in the eel, and that each of them is orthologous to one of the coelacanth and spotted gar *Kissr*. This indicates that 3R did not impact the number of *Kissr* in teleosts, suggesting an early loss of teleost-specific duplicated *Kissr* genes, before the emergence of the elopomorphs (Fig. 5). Apart from the eel, only one or two *Kissr* genes have been described so far in teleosts, indicating additional loss events after the emergence of the elopomorphs (Fig. 5).

The occurrence of these many independent loss events may have led to the current situation of *Kissr* in the various vertebrate lineages. Indeed, some species seem to be more conservative than others, and it is of particular interest to clarify what may have driven the conservation or the loss of *Kissr*.

### Conservation of multiple *Kissr*: the example of the eel

As shown by the present study, the eel is one of the most conservative species among current vertebrates, as three different *Kissr* have been retained. Conservation of multiple *Kissr* may reflect evolutionary processes such as neo- or sub-functionalisation. The comparative analyses of eel Kissr peptidic sequences and eel *Kissr* tissue distribution and regulation may constitute the first steps in the understanding of such processes.

#### Comparison of eel Kissr peptidic sequences

The analysis of the peptidic sequences deduced from the three cloned eel *Kissr* cDNAs revealed a conserved disulfide bridge between cysteines at positions 112/192 for Kissr-1, 105/185 for Kissr-2 and 110/190 for Kissr-3. The seven TMD of each receptor comprise 23 aa, except for TMD3 of Kissr-1 and Kissr-2 which comprise 18 aa and 19 aa, respectively. The pairwise comparison of the three peptidic sequences revealed 60.5% identity for Kissr-1/Kissr-2, 63.3% for Kissr-1/Kissr-3, and 63.4% for Kissr-2/Kissr-3. These low identities are mostly due to differences between the N-terminal extracellular domains (28.6% to 40.5% identity) and between the C-terminal intracellular domains (43.6% to 58.3% identity) (Fig. S6). Differences within the N-terminal domains could reflect variations in ligand binding properties, while those in the C-terminal domain may correspond to differences in G protein association properties and activation of intracellular signalling pathways. Future studies, including recombinant receptors and characterization of endogenous ligands, will aim at further investigating the potential differences in the structure/function of three eel Kissr.

#### Differential tissue distribution of the three eel *Kissr*

**Figure 6:**
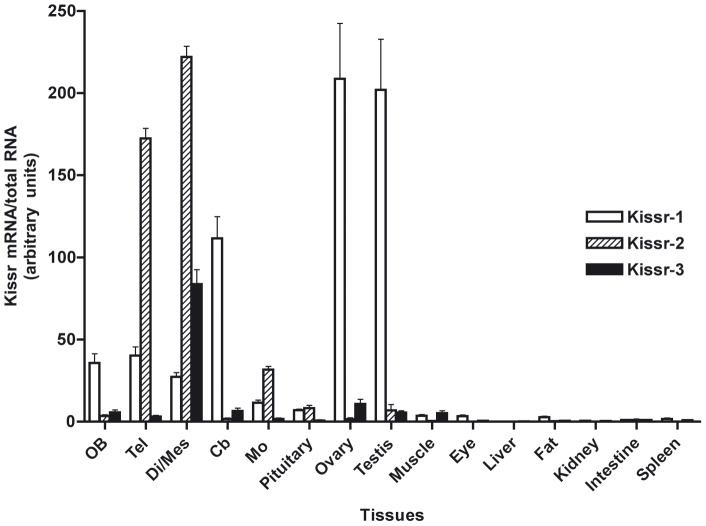
Tissue distribution of the expression of the three eel *Kissr* mRNAs. Olfactory bulbs (OB), telencephalon (Tel), di-/mes-encephalon (Di/Mes), cerebellum (Cb), medulla oblongata (MO), pituitary, ovary, testis, muscle, eye, liver, adipose tissue (Fat), kidney, intestine and spleen. The relative expression of each *Kissr* mRNA was normalised to the amount of total RNA. Each bar represents mean ± SEM from 8 individuals.

Specific qPCR were developped for each eel *Kissr* and applied to the analysis of their respective tissue distribution (Fig. 6).

*Kissr-1* mRNA was expressed in all parts of the brain and in the pituitary. Its highest expression level was found in the gonads, in both ovary and testis. Low *Kissr-1* mRNA levels were measured in muscle, retina and fat tissue, while its expression was close to the limit of detection in the other peripheral tissues investigated (liver, kidney, intestine, and spleen).

*Kissr-2* mRNA was also expressed in the brain and pituitary, with the highest transcript levels in the telencephalic and di-/mes-encephalic areas, in agreement with our previous study [9]. A low expression of *Kissr-2* mRNA was measured in the testis, while its expression in the ovary and all the other peripheral organs was close to the limit of detection.

*Kissr-3* mRNA was highly expressed in the di-/mes-encephalic area and to a much lesser extent in the other parts of the brain. Transcript level was close to the limit of detection in the pituitary. Low expression of *Kissr-3* mRNA was measured in the gonads, ovary and testis, as well as in the muscle, while its expression in the other peripheral organs was close to the limit of detection.

The three eel *Kissr* thus appear mainly expressed in the eel Brain-Pituitary-Gonad (BPG) axis, which highlights the potential involvement of the kisspeptin system in the eel reproductive function. Considering the phylogenetic position of the eel, this tissue distribution could reflect an ancestral role of the kisspeptin system in reproduction, which would have been largely conserved across vertebrate evolution. The three *Kissr* transcripts are all highly expressed in the eel brain, but with various relative levels according to brain regions. Both *Kissr-1* and *Kissr-2* are expressed in the pituitary, where they may mediate direct kisspeptin effects as previously investigated [9]. *Kissr-1* is also highly expressed in both the ovary and testis. Thus, the three eel *Kissr* present a differential distribution among BPG axis that suggests differential putative roles in the control of eel reproduction and implies a potential sub-functionalisation of the three receptors.

In the other teleosts, only *Kissr-2* and for some species *Kissr-3* have been described so far. As in the eel, they are expressed in the BPG axis (grey mullet, *Mugil cephalus* [33]; zebrafish [6], [34]; fathead minnow, *Pimephales promelas* [35]; tilapia, *Oreochromis niloticus* [36], [37]; goldfish [7]; fugu [38]). In xenopus, which presents the three orthologs of eel *Kissr* [4], all three receptors are expressed in the brain, while only *GPR54-1a* (*kissr-1*) and *GPR54-2* (*kissr-2*) mRNA are detected in the pituitary, similarly to the eel. In placental mammals, only *Kissr-1* ortholog is present and expressed in the brain, pituitary (human [2], [39]; mouse [40]) and ovary (rat [41]; human and marmoset [42]), where a role in local control of ovarian functions has been suggested.

#### Differential regulation of the three eel *Kissr* by experimental maturation

Gonadal development in the silver eel is blocked at a pre-pubertal stage due to a deficient production of pituitary gonadotropins, which results from a dual brain blockade: lack of stimulation by GnRH and direct inhibition by dopamine (for review: [43]). Experimental gonadal maturation was induced in female silver eels according to classical hormonal treatments [19]. The expressions of the three *Kissr* were analyzed by qPCR in the BPG axis, which are the major sites of *Kissr* expression in the eel.

**Figure 7:**
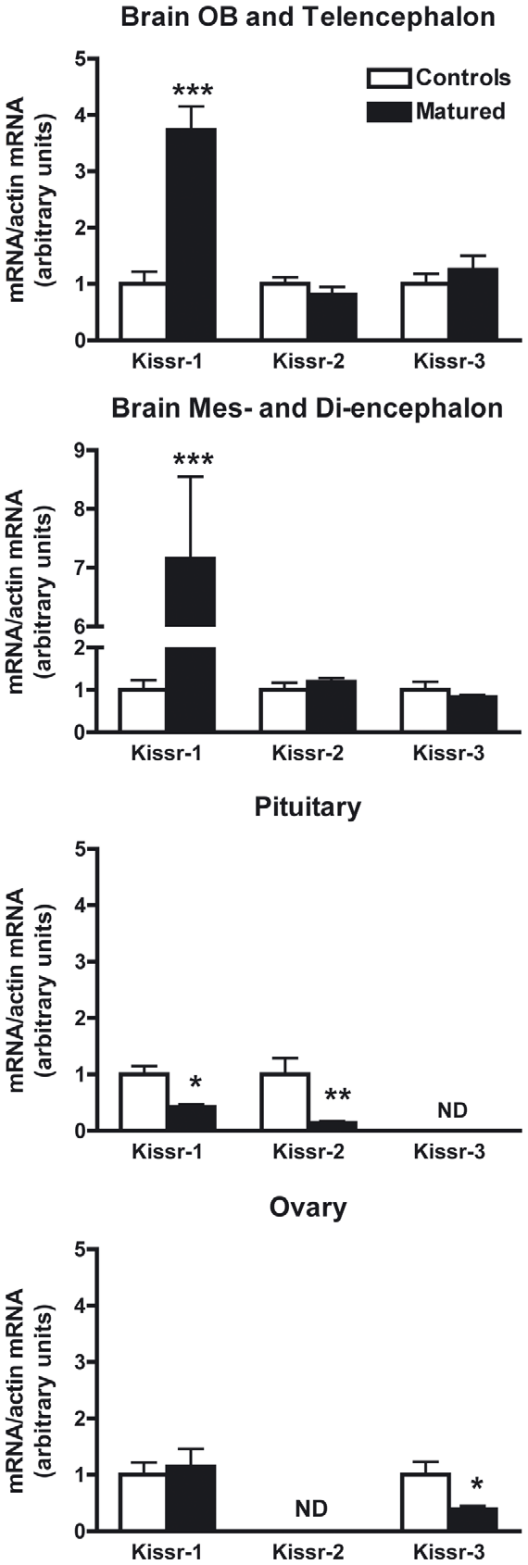
Regulations of the three eel *Kissr* expressions during experimental maturation. The relative expression of each *Kissr* mRNA was normalised to *ß-actin* mRNA. Each bar represents mean ± SEM from 7 control and 9 matured female eels. Significant difference between control and matured groups: *P<0.05, **P<0.01 and *** *P*<0.001 (Student's t test). N.D. = Non detectable.

In the brain (Fig. 7), *Kissr-1* transcript level was significantly up-regulated in matured eels as compared to control ones (x3.73, *P*<0.0001 in olfactory bulbs/telencephalon and x7.15, *P*<0.0001 in di-/mes-encephalon), while no changes were recorded for *Kissr-2* nor *Kissr-3*. In the pituitary (Fig. 7), *Kissr-1* and *Kissr-2* transcript levels were significantly down-regulated in matured eels compared to controls (x0.42; *P*<0.05 and x0.14; *P*<0.01, respectively), while *Kissr-3* remained at the limit of detection. In the ovary (Fig. 7), *Kissr-3* transcript level was significantly down-regulated in matured eels (x 0.39; *P* = 0.01), while *Kissr-1* transcript levels did no change significantly and *Kissr-2* remained at the limit of detection.

We also analyzed by qPCR the expression of brain *mGnRH* and pituitary *LHß*. Significant increases in mRNA levels for brain *mGnRH* (x5.6; *P*<0.001) and pituitary *LHß* (x206; *P*<0.0001) were measured in matured eels, as compared to control eels (data not shown) in agreement with our previous studies [20], [44], [45].

A parallel study was performed on the regulation of the three *Kissr* transcript levels in experimentally matured Japanese female eels. Eels were treated according to Jeng et al. [46]. As for the European eel, a differential regulation was evidenced. This was shown by selective increase in brain *Kissr-1*, decreases in pituitary *Kissr-1* and *Kissr-2* and decrease in ovarian *Kissr-3* transcripts, in matured eels as compared to controls (data not shown).

Those results clearly evidence a differential regulation of the three eel *Kissr*, with receptor- and tissue- specific variations. The selective up-regulation of *Kissr-1* expression in the brain suggests that *Kissr-1* may contribute to enhance kisspeptin stimulatory control of GnRH at puberty in the eel. Similarly, brain *Kissr-1* expression increases at the onset of puberty in mammals (rodent [47]; monkey [48]). This role may have shifted to *Kissr-2* in other teleosts, which lack *Kissr-1* ortholog. For instance, parallel variations in the brain expressions of *Kissr-2* ortholog and *GnRH* were observed during sexual maturation/puberty in several teleosts (cobia, *Rachycentron canadum* [49]; grey mullet [33]; fathead minnow [35]; Nile tilapia [36]). We recently revealed an unexpected inhibition by kisspeptins of *LHβ* expression in eel pituitary cells *in vitro*, providing the first evidence of an inhibitory role of kisspeptin on gonadotropic function [9]. In the present study, we observed a down-regulation of eel *Kissr-1* and *Kissr-2* in the pituitary, while *LHβ* expression largely increased. This suggests that the inhibitory control of kisspeptin on *LHβ* expression could be removed during the sexual maturation by the down regulation of its receptors in the pituitary. *Kissr-3* was down-regulated in the ovary implying a potential involvement of this receptor in ovarian function. Future studies should aim at clarifying the role of kisspeptin system at gonadal level in teleosts.

The regulation of the expression of the three *Kissr* in the BPG tissues reinforces the hypothesis of a conserved ancestral role in vertebrate reproductive function. In addition to their differential distribution in the BPG axis, their differential regulations further strengthen the potential sub-functionalisation of the three *Kissr* paralogs for the control of eel reproductive function.

Our previous studies, including castration experiments and steroid treatments, demonstrated that the activation of mGnRH and LH in matured eel result from a positive feedback by sex steroids [44], [45], [50], [51]. Increasing data support a role of brain kisspeptin system in the mediation of steroid feedbacks on GnRH neurons in mammals (for review: [52], [53]) and recently in teleosts (medaka [54]; goldfish [55]; zebrafish [34]). Future studies will aim at investigating the role of sex steroids in the differential regulation of *Kissr* paralogs in the eel.

## Conclusions

In conclusion, our study provides the first evidence of multiple *Kissr* paralogs in basal osteichthyans, with the cloning of three *Kissr* in a basal teleost, the eel, and the prediction of four *Kissr* in a non-teleost actinopterygian, the spotted gar, and four *Kissr* in a basal sarcopterygian, the coelacanth. Phylogenetic and syntenic analyses support the existence of four *Kissr* paralogs in osteichthyans, leading to the proposal of a new, simplified classification and nomenclature (*Kissr-1* to *4*). The four *Kissr* paralogs may have arisen during the two rounds of whole genome duplication (1R and 2R) in early vertebrates, followed by multiple gene loss events in the various groups of actinopterygian and sarcopterygian lineages. In particular, no impact of teleost-specific 3R can be recorded on the number of *Kissr* paralogs in current teleosts. Sub-functionalisation of the three eel *Kissr*, as shown by differences in their sequences, tissue distributions and regulations during sexual maturation, may have represented significant evolutionary constraints for the conservation of multiple *Kissr* paralogs in this species.

## Supporting Information

Figure S1 Three eel *Kissr* gene sequences. Genomic sequences of the eel *Kissr-1* extracted from the scaffold 90.1 (A), *Kissr-2* extracted from the scaffold 3158.1 (B) and *Kissr-3* extracted from the scaffold 1832.1 (C) (European eel genome [11]). Nucleotides are numbered from 5′ to 3′. The five exons of each gene are shaded in grey.
(DOC)

Figure S2 Molecular cloning of eel *Kissr-3* splicing variants *Kissr-3_v2* and *Kissr-3_v3*. Nucleotide and deduced amino-acid sequence of the cDNAs encoding the eel Kissr-3_v2 (A) and Kissr-3_v3 (B). Nucleotides (top) are numbered from 5′ to 3′. The amino-acid residues (bottom) are numbered beginning with the first methionine residue in the ORF. The asterisk (*) indicates the stop codon. The predicted transmembrane domains (TMD) are underlined.
(DOC)

Figure S3 Prediction of four *Kissr* CDS from the coelacanth genome. Nucleotide and deduced amino-acid sequences of the CDS encoding the coelacanth Kissr-1 (A), Kissr-2 (B), Kissr-3 (C) and Kissr-4 (D). Nucleotides (top) are numbered from 5′ to 3′. The amino-acid residues (bottom) are numbered beginning with the first methionine residue in the ORF. The asterisk (*) indicates the stop codon. The predicted transmembrane domains (TMD) are underlined. The exon-exon junctions are represented by two nucleotides coloured in red.
(DOC)

Figure S4 Prediction of four *Kissr* CDS from the spotted gar genome. Nucleotide and deduced amino-acid sequences of the CDS encoding the spotted gar Kissr-1 (A), Kissr-2 (B), Kissr-3 (C) and Kissr-4 (D). Nucleotides (top) are numbered from 5′ to 3′. The amino-acid residues (bottom) are numbered beginning with the first methionine residue in the ORF. The asterisk (*) indicates the stop codon. The predicted transmembrane domains (TMD) are underlined. The exon-exon junctions are represented by two nucleotides coloured in red.
(DOC)

Figure S5 Alignment of the amino-acid sequences of 51 Kissr used for the phylogenetic analysis. The amino-acid sequences were aligned by ClustalW and manually adjusted. The identical amino-acid residues between sequences are shaded in black and the similar (with similar physico-chemical properties) amino-acid residues are shaded in grey. Sequence references are listed in Table S2.
(TIF)

Figure S6 Alignment of the deduced amino-acid sequences of the three eel Kissr. The entire amino-acid sequences were aligned by ClustalW and manually adjusted. The identical amino-acid residues between the three sequences are shaded in black and the similar (with similar physico-chemical properties) amino-acid residues are shaded in grey.
(TIF)

Table S1 European eel gene specific primers. Specific primers (F for Forward and R for Reverse) were designed for PCR and qPCR amplifications. *Kissr*, kisspeptin receptor; *LHβ*, Luteinizing Hormone β subunit; *mGnRH*, mammalian Gonadotrophin Releasing Hormone.
(XLS)

Table S2 References of the Kissr sequences used in the phylogenetic analysis.
(XLS)

Table S3 Names, references and locations of the genes used in the synteny analysis.
(XLS)
