# Supplementary material for: Multiple Kisspeptin Receptors in Early Osteichthyans Provide New Insights into the Evolution of This Receptor Family
Source: PLoS One. 2012 Nov 20;7(11):e48931. doi: 10.1371/journal.pone.0048931 (PMC3502363; doi:10.1371/journal.pone.0048931)
Supplement: Figure S1 — Three eel Kissr gene sequences. Genomic sequences of the eel Kissr-1 extracted from the scaffold 90.1 (A), Kissr-2 extracted from the scaffold 3158.1 (B) and Kissr-3 extracted from the scaffold 1832.1 (C) (European eel genome [11]). Nucleotides are numbered from 5′ to 3′. The five exons of each gene are shaded in grey. (DOC) [file pone.0048931.s001.doc]

**>eel Kissr-1 gene**

TTGTCCTGCTGCTTGATATTACACACATTCATAGTAAAATGTCCTGTGTTTATTCTAGTCTCAACAGAATGTTATCTATTAAAAGATGAATTAACACTGGACATTTTACTGTGTAAGCCAACTATGTAACGTGCATGTACTACAATAAGGACACGCGCAAGGCATAACAGCCTTTTAAAATGTTTTACGTTCCAAGGTT**ATGCTCGGCGTGCCCCCGAACAGCTCAGGATCCGCTGTTCTTGGTAACGAGTCCTGCAAGTTGGCGAAGCTCTGCAACGATTCAGCCAAATTCGAGCCCCCGTTTCTGGTAGACGCATGGCTCGTGCCGCTTCTCTTCGCTATTTTAATGGTGATCGGACTGGCAGGAAATTCACTGGTCATCTATGTGATTTCTAAACACAAGAGAATGCAAACTGCAACTAACTTCTATATTG**GTAAGAAAAAAGTTAAATTTAACTTCCTTCGTTTCATTGTGTTGTTCTGTTGACGACTATGCTATTCGTAGCCTACAGAAAAGGTCGTTGGCCTACATTGGTGTTGTTTCCTGCTTTTCTTTCTATAATATTCTTCTCCGGAAGTTAAGTTTTTATTTATGTGGTAAAATAAGTTTAATTGGCCTTAATTTGTAATAGCCTACTAGCAGAAGCCTATAAATCGCTCTAGAGCAGGGGCTACCAAACTCCCCAAAATAAATAAATAAAAAATTCTGGCACATGCACCTGGTTGTAACAAAACCAGCATACACATGGGGGCCCAGGACCGAGTTTGGGAACCCTACTCTAGAGCGTCTGCTAATTTAATGTAATGTCATTGAATATGCAACTCTGTGTTTCCCTTTAATCTGATCTGAAGGAATATCTTGTCTTCCAG**CTAACCTGGCAACCACTGACATCATTTTTCTGGTGTGCTGTGTTCCATTCACTGCCGTGCTGTATCCGCTTCCCAGCTGGGTCTTTGGTGACTTCATGTGCAAGTTCGTCAGCTATATCCAACAG**GTCAGGAGCCTTTATCGTAATTGCTACATTGGTGTTGGCTGCAACTAGGCGAATGTTATAAAATTATGCAATGCTAGTTTTATTAGCTGTTCTTTTGACTTTTCGTGCCATATGCCTGAATGACAGCTAGATGGAGGCAGAGTAATTTAACTTCTATTAAATTGATATTACACACATTCATAGTAAACTGTCCTGTGTTTATTCTAGTCTCAACAGAATGTTATCTGTTAAGAGATTAATTAACACTGGACATTTTACTGTGTAAGCCAACTATGTAACGTGCATGTACTACAATACAATACTATCTCTTGGACAGGCTTTGTGACTGCACACAACACAACAATATAGGTGTTTGTTTACGCATCCCTTTACATACATGTGCTGTCAATGGGATTTGTTAAAAATACATGCAGCTTGATCTTTTGATTTTCTTATAGAAAGCACGAGGCTATTAATTTAGCATTGGAATCAGTACAAGGATTTGAATTTATATTATTTTAG**GTGTCAGCACAGGCAACCTGTGTAACACTGACAGCAATGAGTGTGGATCGTTGGTATGTCACTGTATGCCCACTGCGTTCCCTGAGTTGTCGAACCCCACAAGTTACCACGCTGGTCAGCATTGGAATCTGGATCG**GTAGGTTACCAACTTGTAGCACAAAAATCATTTAAATGTTTTGCTGATGACTGTCTCCATCATCATTACATGTTATTTTTCATCTGGTCAGTTTCTTGTTTGAATCATTCCATGTTTTTATATTCCACTTACATTGTAGGCTATGTTTTTGGTTTGCATGCTTTTGATCGGTTTTGCCAGAACTAAGGAAGATGTTTTGTTTAAAATGTCAGTTTTGGTTGTGTACTGTTGTTCCTACTGTAATATCTTAAGACATCGGGGGTGTTTTACATACTTGAGTAGCAAAGACTTGAGTGTTGAAATTTGAATGACAGTGAAGATGAATGAAAATAAGAGGTGTGTTGATGTTATTAAGTGTCAACCTATAGACAAAACCTAAATAGATCAAATCACACTACACTGAATATAGGCTTAGCTCAATGATGGCTTACATTAGAATACAGCAAGTGTTGATTCTAAACTCTGTGCACTGTAGATGGAAAATAACACAAACCAGTGTTTATCAGCGATAACTTATGTTAACATGCCGTTTTTTTTTAATCAACACTCATACAATATTAAAAAAAAAATAAATAAAAAAAATAAGTGTAGCAAATTTGTCAGACTGGGGGCTTAAACCCTGGTCCCCCATGTGGCAATCAAACACCTAGTCCACTGCGCTACCAGAGACTGGAGGTGACTCAAGTGCAGAGCTACACCAGCAGGAGTTCAGTTGGTTTTAATCCACAACCAAAAAACACAAATTCAAAAACTCAGAACTAGACAAAAAGATAACACTCCTAAGCAGGGAAAAAGTTACAAGCAGGCAGAACAAGTCAAATACATAATCCAAAAATCAGAACCCCAAACAAACAGAGCCAAAGTCAAATACAACAGATCAGTCCGTGAAACAAGCAAACACACCAGCAAGGGAAAATCCAAAAGAGATACCGATAACAGTCCAGGTCGAATCAGGCAAACTATTCAGCGGGGTCAAAAAGGTGGCTTGAGCTTGAGAAAAGCCAAGGAATCATAAAGACTTAACAAAGGCATAGACAGAGGACCGGGCTTAAATACACTGAGAAAACAAGCAAACAAGGCACATGACAGAGACAATGAGGTACAGGTGGGCAAGTTAACAAGAGGGCTGGGTCAACATAACAAGGGAGGCTAACCAGTGTCCAGAACAACAAAGAGTCTTTGGCACCCTCTGGTGGTTAAAAGGTAGTCCAACCCGTGACAAAATTGTGGCAATATACTTAATATAAAGTGTTCAATTTAACAGAAATTGTGGTGAATATGCCCCATGTTGCTTTAATATCAATGCCTTACTTCTTACTCTATTTTGTGACAAAAATGTGATAAATATAAATGTATTGTTTAGGAAATCATTCACTATGCTTATACACCATTTAATAAATGTTTGAGAGACTTATTTCAGTCAAAATTTAATAAAATATGATATAAACACCCAAATTATCACATTAGTTTCACTAGTTGACTAACACTTTTTAGTATTATATTCATTATATTCAAATGAACACTTGTATTCACAGATAAGGATTGCTATCCAAAGTTCTAAACTGAAGTAATCAAACAATAAGTAAAAATGAAATGCTAAAAATGACCAGTTTTTTTGTTTGTTTTTTACTTTAACCACTATCTAATAAACTGACTAGACATGAAAAGTAGAAAAATTAAATCAGATCATCCAATGTTTGCGGTTTAATAAATTGCTTTTAAAACTAATCAAATATCTGTCCTGCGTAAGTGTTTATGTTTTCCTCTCTCTGTATCAG**GCTCGTTCCTGGTGTCTGTGCCAGTGCCAATGTACAGCAAGACCATGCTGGGTGAGTGGTATGGTCCCCAGGTCTACTGCACAGAGAATTTCCCCACTCTTTTCCACAAGAAGGCTTTCATTCTCTACAACTTCCTGGCTGTGTACATGCTCCCACTGGTAACCACCTGCATCTGCTACATGGTTATGCTGAATCGCATGGGTCACACCTTTGTGGAGCCTCTTGAAAACAACCACCAG**GTACCCATCAGTCACTTAAGAGCACATTTGAAGGAGTAAAGGCTCTACAAATATTTGGCCTAGATGCAGGGTGCCATATGTTTCCATATATTTGATAGTTTTTCATTAAAATTTCATTAAATTGAAAAAAAAAATATATATCAAGGATTGGTTGCTGTTTCTTCTAGATATTGTAACCTTTTGCAAACACATATTTGGGAAAAATACCAATTGTAATGTTCTAACTTTTAATTGAAGATAGCTCCTCACATATTAATGTTAATGTTTAGCTTCTATAATGAACTGCTTAGGAATTGCCAGCCTTAAAGAGTGCTTATCTGGACTGTCTGCAGAATTTGGTGAGTAAAGACAAAAAAGTTTTGTCAGGTCTTTTCCTTTGTCTTCATTCATCACTCCTTGCACCTCAG**CTGCAGGTCTTGTCGGAGCGCTCCGTGGCCATGAAGACCAAAGTTTCCCGCATGGTGGTGGTGATCGTGCTGCTCTTCACTTTGTGCTGGGGCCCCATCCAGTTCTACATCCTTGCGCAGGCCTTCAGTCCACACTTCCGTCACAACTACTGCACCTATAAGCTGAAGATCTGGGCCCACTGCATGTCCTACACCAACTCTTGCATCAACCCCATTGTTTATGCCTTCATGGGCGTCAATTTCCGCAAAGCCTTC**AAACGAGCTTTCCCTTGCATTTTCAAACAGCGTGTGGCAGTTGCCCAGCCAGCACATGGCACTGCCAACACTGAAATGCACTTTTTCTCTTAGACCTCCTAAACTAAGTCTGTTGGTTTCTTGTGTGCTTAAAGATTTTCCTGAGACAATCTGGCTCATGACCCCAAACAAGCTGGGACTGGCTCTGTTGACCTC

**>eel Kissr-2 gene**

TTTTTTTTTTTTTTTTCTCTCCTTGATTCCGGGAAGATCCTGTGCGGCATGGGGCATGCTCTGACGTTTCTGTCCGCTTAAGGATTTTGTGAAATTACTTAACACCTTAATGTCTATCTGCAAGTCGAAAGGAGCGGACCTTTGCTGATTGCTAGCCATATCTGCATTTAAAACTCTTCATTAGCTCTTTATTAGTTTTTTTTATTTCTCCCACTGAA**ATGTGGAATGCCACTGAACTTCTCTTCAACCTGTCTGAGGTGAACGGTTCCATGGACGGAGGTGAGGATGGGGAGCATCCGTTTTTGACGGACGCCTGGCTGGTGCCTCTCTTCTTCTCTCTCATCATGCTGGTGGGACTAATCGGGAACTCCCTGGTCATCTATGTGATCTCCAAACACAGGCAGATGAGGACCGCCACCAACTTCTACATAG**GTGAGTGTGAGACTCTGGCCGTAAGAATCCTGTGTGTTTGTTTTCTGATGTTTTATCGCACTTCCTGGCATNNNNNNNNNNNNNNNNNNNNNNNNNNNNNNNNNNNNNNNNNNNNNNNNNNNNNNNNNNNNNNNNNNNNNNNNNNNNNNNNNNNNNNNNNNNNNNNNNNNNNNNNNNNNNNNNNNNNNNNNNNNNNNNNNNNNNNNNNNNNNNNNNNNNNNNNNNNNNNNNNNNNNNNNNNNNNNNNNNNNNNNNNNNNNNNNNNNNNNNNNNNNNNNNNNNNNNNNNNNNNNNNNNNNNNNNNNNNNNNNNNNNNNNNNNNNNNNNNNNNNNNNNNNNNNNNNNNNNNNNNNNNNNNNNNNNNNNNNNNNNNNNNNNNNNNNNNNNNNNNNNNNNNNNNNNNNNNNNNNNNNNNNNNNNNNNNNNNNNNNNNNNNNNNNNNNNNNNNNNNNNNNNNNNNNNNNNNNNNNNNNNNNNNNNNNNNNNNNNNNNNNNNCTAGTCTGTCTAGGTTTGCACACTCGATACGACGTTGGATGTTTGCACTTGGGTCAAACATTGTGCAAACGTTGTGCAAACAAACATGATATATAGTTTACAGAATGTAGCTTGGCAGTGGGGTTTTCTGGAAATCAGGCAACTGGGAATGCTGGATGGGGAAAGAAAAGCAATGTTTTGATGTGATTGAAAGATGAAATATAGGGCTTGGCTATATTGGCTAGTGGGGATGATGAATTATTCTTGGGACTGAATACGACAGAAAAAATTATTGTAAATGAGCTGGAAGATTAACTGTTCAAGGATCTGTGTAGAAACTGCACACTTTCAAAAATAAATAAATAAATAAATAAAAAAAGCACTACGTGGCATATGTTTTGCTCCATCTACCTGCTCCATTTAGTCAACAAATCACCCAGCAATGGCTGAGCAAAGCCGGTCTGTCACACATGCTGATTTTTATTTATTTGTATTTTTTGAAAAGCAATATGAGGATACAGCCTTTTCCATCAGAGAAATGGGTGTAGACATGCTCTATTTTCCAGCTGTGAACCGTAATGTGTTGGTAAACAGAGGACAGAGAAATCACAGATTTTAAATGGAAACATGCTGTTTTAAAAATACATGCTGACGTCTTTTCCTGGGGGAAAATAAGTTCTGTCATTTTCAGTTTTTGCCTGCAAGCACTCTTGAATTGTGAGACATTTGGACTTTTTTTCCCCTCTCAGTGATTTTGGTGGCTGGTCTATGTGAAGATTTATAAAAATTTGTCCAGAAACTTCCATTTGGGCAAATCAGAAACATCACACACCCATTTTCGTTCATTGCAAATAATGATTTCCAAGTTTTTTGCCCAGGACCCAGTCAAGTCAATATAGGATAAACCACACAAGTGTTTGTACATTGCTTTTTAAATGTCTCATATTTTGCTAAGAAAGATATGTAATTAAGAGTAGAATCCAACCATTACAGTGGACATGCTAGGAAGTGAGGTGTTAGCAGATGTTTTGGTTGTATGAAGCAAACTCTATCAATTATTTATAGATACTTTCCCTACAGTACGTGACATTTTTAATAAGTGGATAATCATATGTTTGGTTAGACCACCGATTCTCAGCCTTGGTTTTTGGGCCACACCTTTACAGTATGCTGCATTTCGTTCTGGCTGAGCTGTTAGTCAATTCTGTTTCAACAAACCTTTCTTTCTTTTTTTTTTTTTTACCCAGATTTATATTTTCTCTCAGTTTGGAACACCAAAACATATTTCAAAGCACATTTCACCCTTCCCCAGTCAAGCACATTCATCAAACCCGTCGACTGAGCGTGTTGTCATAGCACCAGAGTACAGAACACACGCCTGATCAATCACAGGAGTCACAAAACCTAACCTGGCTGATTTTGTATGCCCTTAACAGACAACTGGGATAGGCAAATTCTGAAAGTTTTTTTTAGTTTTTTTTTTTTGGAAATGGAACAAACTGCCCTATATTATAAATATGTGGGTTTTTTTTGATAATATAATGATTAATGAAGTAATATATTGCAACATCTGAAGGAGCAAATAATATGTACATTTACCCATATAGTGTGAAGAGTGGTAATATCCTGATAAATATATTTAATTTCAAAATCAGTTTGTAGACAGGGTTTCTCAGTCTAATGAAGGGTTCTGGACTAGTAAGACCAGCTTTGTGGAATTTTATGAAAGGATACTGTAGGTTCTGTTTTCCAGCATTGGTGCTTGTGAGCAGTGCTGTGTACTTGGACCCTATATAGATATAGATACGTATATATAGAGATTCTGAGCCACAGCTTCCTTTAATGTGTTTGTTCCCTAACTGTATGCTCCTCTCCAG**CTAACCTGGCAGCCACAGACATCATCTTCCTGGTGTGCTGTGTCCCCTTCACTGCCACACTCTATCCCCTGCCCAGCTGGATCTTTGGGGACTTCATGTGCAAATTTGTCGCTTTTCTCCAGCAG**GTATGTACTGTAACACTAATCTGATGCAACAAGATTGATGATTGGACAATGTTAGAAACCTTGATCACTACTTGATCCTCAAACAAGATTCACTATTAGTTATCACAGATGGAAATTTACTGAATCAAAATGGATCAAGTGCTGTCTCTTATCCATGGACCATAGCCTCAGTGCCCTACAGGATCCATAGCTGTAGCTGTCCATCGCTGTCTTCTATGGGTAGTGATTTCTGAACTGTCTGCTCAATGCACAAAGCAGCAATCAATTTAAAGAGATAGCACAGCTGAACTATGATGGTATTCTGATGATCATTTGTCTTCCTCCGCATCAATCCAATCAACCATCCTCCTCCTGCTCCTCCTTGTTCTTGTTCTTCTTCTGTTCCCTTTAATTTCTTCATCCTCTTCTATCAAAGCCCAAAATGCGGACAATTTGGATGACTTAAAATATATATGAGTAAAATATTGAAGAACTAAATATTTAATTCAACATATCACATTCCTCTGAGCCCACCTAAATCAAACTGTAATGATTCGCTTGTGATTTTCCAGCCCAATATTCTCTGTCATTATTAAATAACCAGCAGATCAGACACATTTGAAACTGTTGAGATTTGAATAAAAAAAATTTGCATTTCAGAGCTTGAATGGGAGCACCAGTTTCTACTTTATATTTAAGGAAGGATTAAACAGTACAGTTTTATACTGGTACTGGTACTGGTATATAAGCTTTCTAAATTCCATTCAAATTCTTTGCTCACATATTTTTAGGTCAAAACCAAAACAAAATTGATACGCATCGATTACACATTTTTAATAATAAAATAAGTAAAAATTGATATTTTGAAATATCACATTTCAATGTCTCGGTCATCTCTATATATTTTGTTCAATGTTGATATTCAAACAATATTATACTTTACTCACACTGAAGATTGACCTTAATATCTGTCATTTTTCTGTATCATATAATAAGCCTTACAGATATAGTCAGAGGCAATTATAAATGTTTTTCATACAGAATGTAATCATCAGTTTCGTTTAACTAATTCAAAGACTAGACTGAAATTTTGTGATATAAATTATAAATTGAATGTAATTTCTCCTTTATTGTAACTGAAACTTGGCTGATGGGAAGATAAAAAGTTAAAATTACAAGCTGGGGAACTCAGCAACTTGGGTAAAAATTGCAACAGCAAATAATATGATCTCTTGAACTCTCTCTATGTCTCTGTTGTGGCTGATTCCTGAGTACACAGGAAGACACCACTTATACACAGACAAAAACATTTAGTGAGTCGTCTGGACATCTTCAGGACCCACTACACGCAGTAGATGTTCTGATTGGCTAAAATAAATAGCACTGAACCATCACACTTACTATTTAACCTTCTGCCATCTTCAACGTTTGTACTTAAAATGTAATTAATTTATAATCTTTTTTTTTTTCCTCAGCTATAGGCTTTGTCAAATTATTTAATTCTCTGCTAATACCTTTTGTGGCAGCACATTTAAATTGAAATGTTCTGCTTTGCACAGGGTTGGGAATAGACAAAATACAACTGGAGCTCTGATTGAGGCGATTGTACAGGCATCATAATTTGCTTTTCGTAATTCTTTTTTTCTTTTCTGATTTGACTCACTAGAACATCCACTGGACCATCAGTCATTGTAATAGGTTAATGACTGCAGGTCATTTCAAATTTGTATTTCAAAGGGATTCAATTCAGAGAAGAATGCTTTTCTTATAAGTAGGCTCTCAGCTAGACATGATGGAAGCCTTCAGGCCTCAAAGGGCATTAGGATAGCTCTCAAAGTGCATGATGGGAGAACATTTAACTTACCAATTTAAATAAATCTTTGACTCCTCAAAATACTTCAAATTGTAAGTTTCATTTTAATGACTGGATTGCAGAATACTTCTCTCTAACTTTTATATGTTTTTATAAAAGGTTCAAAGCTGGAGAAAGGGTAGGCATTAATGAATTTGGCTTCACCAATTTTATATAAAATTAATTTTACAGAAGGTATGTTACCATGGGAACTCGTCAACAGCCCTTTTCCTGATCAGCGGTCTCTATATGGCATATGCAAACATATTACCAGCACTGGGGTATTTCATTAGGCAGAATCAGACATTGTGTNNNNNNNNNNNNNNNNNNNNNNNNNNNNNNNNNNNNNNNNNNNNNNNNNNNNNNNNNNNNNNNNNNNNNNNNNNNNNNNNNNNNNNNNNNNNNNNNNNNNNNNNNNNNNNNNNNNNNNNNNNNNNNNTGTGCATGTGCGTGCGCATGCGTATGTTGGGGGGGCTAGGGTTGAGTCCCTGCTAGGTCTCTGCTGTTGTATCATTCAGTAGAGTACTTAACCTGGATTACTCCAGTAAAACACCCAGCGGCACTCAGCTTGATTATTACTGGCTAGAGATGAAAACTAGCCTTCCACAGGCACGGCCATTTTGTTTCACTTTCTGACAGCTGAATGTTGCAGCTGCTTCTCTAACGCAGGATTCCTCTAGGCCAGGGATCTCCAAGAAACACCATGTCAGTTTCTGAGTTTCATGGAAACTTCCATTATCTATACCCGCTTATCCTGAGCAGGGTTCGGGGTGGTGCTGGAGCCTATCCCACCGTGCATTGGGCTAGAGGCAGGAATACACCCTGGACNACACGAACACGGGGAGAACATGCAAACTCTGCACAGAAAGGCCCCAAGCCGAGATTCGAACCCACAAACTTCTTGCTGTGAGGCGANNNNNNNNCCCACTGCACCACCGTGCCATCCACTTTTTATTCATTAATTATCCCAATCATTTAGTAATTGATATTTTAGTTACAAGACCGTTCCTGTGTCCCCAACATGAAATATCTTATTGTGGTCTAATCTACGGCTAAACCAGCATAAGTGTATTCAGCCAATTATCTAATTTAGCCAGTGTAATTGGTGACAAAAAAAAATCAGCACCCATACACACCAGCTCTGCTGCAGTGGCATTGGCCACCGCTGCTCTCGACCAATTCTTTTCCTCCTATCTTATCTTCATTTCTGGGGCATTTAAAATTTAAATAGACACAGCCTTGGCTGCAAGTTCAGGAAGAAGACATTTTAAAGACACTGCCAGGACCACAGCAGGGATTCCACCCAACACCTTAGCAGTCTCGACTTCTTTTATCTGAATCTTTTTTGGCCTTTAGTCTGAAAATATGTTGAGCTAAAGCTGTCGGTGCAAAGACTGTCAAGTACTTCAAATTCTGACGTTTTCAGAGCTGCAGTGCAGAGGTCTGTTGTTCTACGATGACATTGCTACTCCCTCCCCCCTGTTAAGAATACTGTATTGTAGTCACGTCTGCCAGATGTCTGAAGATTGAAGCGGTTCATAGTTAATATCCTGAGAAGATCTCTTACAAAAATCAGGTTGTTGAGTGAGGTGGGCTTGGTGGGCCACCAGGGGGCAGTCATCCCTCTGAACCAAGCAGAAACTCTCTGTACAGTCACAAGGAGACGTGTCTTGGATGAGATTCTATACTGAGGCCCTGATTCGAAATTCCAGGGCACCTATTGTGAGCAATGAGTATAGGGCTTAAACCGGCGGTGTCTTTGTCAAACTGAATTACAGGGGCTTCGATTCTGGCCACCTGACTGACACTACAATCCGATTTGGTGCTTTGCTTCTTTGGGTTTTTGCAGCAAATGAGAAATGAATTCTAGGATCAACAAAAAGGTTGACACGCTGAATACTGGAAGATGGGTTACCTCGGGGAGCAGAGGAGATAGATNGTGGGGGAGGGCAAAGTGAATTTCTGATAATTTGATGGGAATGACAAATACTCAGTCTTAGAAGGTTCAAGTACAGGTGTAGAGCAGAACATGGAATGAGAACCATATGCTATCCAGGAGTGGACAGCTGCTGCAGTTGAGTGCTAATCAATACATTTTTCGGAAGCACCTGCCCTTCTGGGCAAATCCCATTCAGAAGGAGATTCGTATTTTTTATGGGTAGTACATAGCTATTAGATAGCAGTGAACAATTTTAATAAATACTTGTGAGAGTGGGCAGTGTTAATTATTTCAACGGGCTGGAATTTTTCTTTTTTTCCTTTCCGGAGTTTTGCATACGCTATTCAGTCATTGCTCTAGTTTAATTAATAGCCAGCTCCCACACACATTCAGATTTCACCGTGTCCTTGCGTGATGGAGAGAAAGAATAATGGACAAAGCCTCCCACCAAAAAAGTGGAAAAAAATAATTAATTTGGAGGCTTTGTTCTTTTCTTCGGCACCATGGCACTTTAAATATGACAAACTATTCAGTATTATACGGCTCAGTGAATGGTTTCCGTTGCTTTAAAACACAGTGGGCAACACATTAGTCTCATGCTGTCTTTGCTGTAGTCCTGGTTACTATCTCTAGACTACATTTCCCTTTCCCTCTCAGCTTCCATTCTCTGTGTTTTGAACTTTGAGTAGAAAAAAAAATGTGTCACTTTCCGTACTGTATGAATGAAGGCACCTTTATACAACAAAAAAAAAACCTTTCAGTGATGGCACCCGTCCCTTCCTGAGCACTCCATGAGAAAATCATTCTGCAATTAAGCAGCCAGTGGTCGGGGTCACTGGAGCCTGCCGGTTGTTGTGATTTAAAAAAAAACAGTATGCCTTGTGGGAAGTGTAGTTCCGAGGCGTGACGAGATCGCGCCGTTCCAG**GTCACCGTCCAGGCCACCTGCATCACGCTGACCGCCATGAGCGCCGACCGCTGCTACGCCACCGTCTACCCCCTCAAATCCCTGCGCCACCGCACCCCCCGCGTCGCCATGATCGTCAGCATCTGCATCTGGATCG**NNNNNNNNNNNNNNNNNNNNNNNNNNNNNNNNNNNNNNNNNNNNNAATAAATAAATAAATAAATGCCAGCTTTTGAATCCAAGAATGTAACAGAAATAACTGGGGGAAAAAAACGTGCCTGTCCGTTTTAATTAAAGGGAAATAGCTGTCATTCCAGCTAAAAGCCGAAATTCCCCTGTGCTCTGTTGGTGAACAGTAGGCCTACAAATACACATCTGGTCATTATCTTTTGTGCACATACCTGACATATTTTCCTCTTGAAGTATTTCAAATGGATATTCACATAACGTAACTGTCTGCGCATCACACATCATTCTATTTATCGCAGCACTTGAAAGGTGTTGCATGTGGGCAGATGGTGATTTTTAGGAGCTAGTTATGTACTAGACTGGACATTAGCCTCGTGTTTCTCTAGAAAATAAAACATATTCCAAATCTGTATTGTTTTTTGTCTGAACTAACAAAAAATGCCTTGCTATCATGATAAACTTTACTTCACTTAAAAACACACGGTCTCTTTTTGCTTCTAATAATGCTAGTACATTTATGTTGAAGATTTATGCATTTACTACTCCTACAGTAAGTCATGTGTTAACAGCTAATATTGGTTCTGACCCAAATGACATTGCAGTAGGATTTTCTTATGCATTTTTGTGTGCACTATTGTAAGCATCTGATAAATGCTTAAAACAAAAAAGCAAGGCGGCATCATATTGGTTGTCGCTTTCTTTGTCCTGACTGAGTCCTTTTTGGTCAAG**GGTCCTTCATCCTGTCCACGCCGATCTTCATGTATCAGCGGATCGAGGAGGGCTACTGGTACGGCCCCAGGCATTACTGCTCTGAGGAGTTCCCCTCCAAGGCCCACGAGAAGGCCTTCATCCTCTACCAGTTCATAGCCGTCTACCTCCTGCCCGTTCTCACCATCTCCCTCTGCTACTCCTTCATGCTGAAGAGGGTTGGGCAGCCAGCAGTGGTGCCCGTGGACAATAATTACCAG**GTACGGAGGTTAAAATGGCTTCCCCCAGCTGCGTTTCCTTTTTGCCTCCTGCTGTGCTTTCATAGGTTGGATAAACAATTTCGGTTTGAAAGGGTTCCGCTTTTTCTTTTTGATCGTCAACATTAAGTCCCTCGTTGACAGTATTTAATTTTATTTACTGTGCCGTGGACACGGAGTGAGGAAGGAAAAACTGCTTTTAATCACAAAAGAAATTATGTCTTTGGTGCCTTTTATGTCTTTATGTCTTTGTGCCTTTGTAAGAAATTATGGAAAACAGTAAAACTAGACAAACAGTTTCACTCATATAATTCGAAAACATGTTGAATGGTCATTTTGTGGAAGTAATTAAAACTTTCTGAGATCATTCTCACTTTAAATTTGTGTATACACGTATTTAGAGTTTGTATTACCCTTGCTGCGTGTATTGTGTTGTTGTGTAACCAGGTTTTATATTCTGTGATGAACTGTAATGCTGCTCAGCTTGCAAAAGAACTTTTAACCTCCCTGAGTCTAACCTTGTTAAATAAAAGCTTTACATAATAAATTTCTTTTCACTTTTTCATAAAACATAATCGAACAATAGAATATTCAAACTCGAGCAGACAAGAGCTGGCCTTTCTGCCAATCACGGCTCATCTGGGCATTCACCAATAACTAAAAAAAAACAAAAAAAAACTTATAATTAAAACTATAATTAAAACAAATTCAGTCGGTCTGATCCAGTCAATATTTTTACTTAACTTTGTTATTCTGTATATCAGTGTTTATATGGACATTTTTGTGTTGATATGTGAGAACCTTTGAGTGAAACTGCANNNNNNNNNNNNNNNNNNNNNNNNNNNNNNNNNNNNNNNNNNNNNNNNNNNNNNNNNNNNNNNNNNNNNNNNNNNNNNNNNNNNNNNNNNNNNNNNNNNNNNNNNNNNNNNNNNNNNNTGAGAACCTTTGAGTGAAACTGCAATTGAAAATCACACGGCAGACAAAAGGCCGGGAGTAAACTTTTCAGTTTTCCCTCTTGCAG**GTCCATCTCCTCTCCGAGCGGACCATAGGCATACGGAGCAAGATCTCCAAGATGGTGGTGGTGATCGTCCTCCTGTTTACCATCTGCTGGGGCCCCATCCAGCTCTTCATCCTGTTCCAGTCCTTCTATCCCAACTACAAGGACAACTACACCACGTACAAGATCAAGACCTGGGCCAACTGCATGTCCTACGCCAACTCCTCCATCAACCCCATAGTCTACGGCTTCATGGGTGCCAGCTTCCGAAAGTCCTTC**AAGAAGGCCTTCCCCTTCCTGTTCAAGCAGAAGGTGCGGGACAGCAGCGTGACGTCCCGCANNNNNNNNNNNNNNNNNNNNNNNNNNNNNNNGGGACAGCAGCGTGACGTCCCGCACGGCCAACGCCGAGATGAAGTTCATCGCCACCGACGCCACCGGCGAGCCGCAGTGAAGGACAGCCGAACAGCAGGTGCCGCTACAGTAAGCAACGCGAGGCCTCTCTGGGGTTCCGTTAGGCTGGAAACACAAAACACAGGCCAGGGCATTGTGGTGAATATTGAAAGG

**>eel Kissr-3 gene**

ATATTAAAGGCAGGGGACAGACTGGTGCTCCTGCTTTCCACCAGACCGCAACCCCATGGGCTGCTGCCCCACGCTGTGATTGGCTGGGACTCTCAGGCTGCTGCAGGCTCAGTTCTATAGGCCTGGACTTGCCGTGATTGGCTGGCATTGTGAGGACGCCGGTTACC**ATGGAGGACGGATGGCAACCGAACGGCACCGCCGCTCCACCGTGCGACCCGGACGCGGCGTGCAACGGCTCGGCGCTGGCGCTCCTGACTCCGCCCCTGCTGGTGGACGCCTGGCTGGTTCCCCTGTTCTTCGCCATCATCATGCTGCTCGGCCTTGTGGGAAACTCCCTGGTCATCTACGTGGTCACCAAGCACCGGCAGATGAAGACGGTCACTAACTTCTACATCG**GTACGAGCCGCGGTCACTGCTAGTGCACATCGGTGTATCGGGAACGGGTGGGCGTGTCTGCGGATTGATCCGGTTCTGCCAGCGTTCAGTTTGAATGGCTTTACTAAGCAACCATCAGTCGAGACATGCCGGTCCTGGTGGGGTCTATCTGGGCTGCCTGTTGCGAGGTTTTCTTTGTCATTAGAATTCACACTCTCTCCACACTGTCCTGCAGGGATCGCAAAAAGTCAACAGTTTCTTGTGTACATTCTTTTTCCTTTTTATTTTTTATTTATAGCCATATAAAATCGTGCCTTACCCTATAGGAAATCATGTAACATTATAGGAAATTCTCAGGCAAATTTTTATTGTAGTTTTTTGCATGAGCACCTTGTGGTGCCAGCTACCTTTATGAACAGTGTGAATTGTGTCCATTGTTATCTGAGCCTCCGACTGCAGGTTTTTCCTTTAGGGCTCAGAGTTGTTTGTTCTTTCCAG**CGAATCTGGCAACCACTGACATCCTATTCCTGGTGTGTTGTGTTCCCTTCACTGCGACCCTCTACCCTCTGCCCAGCTGGGTGTTTGGGGACTTTATGTGCAGACTGGTCAACTACCTTCAACAG**GTAAAACAGAACTTCACCTCAATATCATCATCTCTTACAGGTACAGCTGACTGAGGATAGGATAATGTAAGCATGGTGCAGGTACAACTGACAGGATGGGATGGTGTAAGGGTGGTGTAGGTGTAGCTGATGGACGATATTGGTGGTCTAAGGGTGGTGTAGGTGTAGCTGATATAGGATAGGGTGGCATAAGGGTGGTGTAGGTATAACTGACGGAGGATAGGGTTGTGTAAAGGTGGTGTCAAGGTGATGGTGTACAGGTGACCTCCTGCAG**GTGACGGTGCAGGCGACCTGCATCACGCTGTCGGCCATGAGCGTGGATCGCTGCTACGTGACCGTTTATCCTCTTCAGTCCCTACGCCATCGAACGCCACGCATGGCCATGGCCGTCAGCATCGCCATCTGGATAG**GTACCCCACCGCTTACCCACAATGCCACTGCTTACCCACAATACCAGTCAAACACAACGCCACTGCAGGTGTATATACCTGACCTGCACACGCACATAATCACACGTCTACATCTGGCGTGATCGAAGTGAGTAAATATGCCCTGGAAATTACAGCAATGTGACAGTTATCGATTGGTGTTGCGTGCTAGTGTTTGTTTTAGGGTGTGGATTGTGTGTGTTTGGGTTGGTGTCGTGCGTTACGGTTTCGTGTTAAGGTTTGAAAGTTTGGGTCATGTGAGTTTGGGTCGGCGTTGTGCGTTAGTGTTTCTCTCAGGGTTTGGGTTGGGTTGGTGTTGTGTGTTACTGCTTTGGTTTTGGCTGTGTGAGCTTGGTTTGGTGTCGTGCGCTAGCAGCTGACATGTTGTAATCACTTTCTACTCACCAACTGGCAGTGTGCCCGTTATTGAGCAATGTTCTTTGGGTTTTTGGGTGGGGCTTCACATGGGGCGGGGGGAACCTCTCCTCACACCTCGTGTCTCTGGCAG**GGTCGCTGGGACTCTCTGTGCCTGTGGCGGTGTACCAGCGGCTGGAGACGGGGTACTGGTACGGCCCGCAGGTGTACTGCACCGAGTCCTTCCCCTCCCCCGAGCGCCAGAAGGCCTTCATCCTCTACACCTTCCTGGCGGTCTACCTGCTGCCGCTGCTCACCATCTGCCTGTGCCACGCCTTCATGCTCAAACGCATGGGCCAGCCCGCGGTGGAGCCCGCAGACAACAGCTACCAG**GTCAGCGCACGCCCGTGCACTGTGTGTGTGTGTGTAGGTGTGTGTATGGGTGTGTGTGTGNNNNTGTGAGAGTGTGTGTGTTTGTGTTAGACAGAGAAGTGGTGTTTGTGTCTGTCTGTGTGATCATGTGTGTGTGTGTGTTTGTGTCTGTCTGTATAATCATGTGTGTGCGTGTGTGTGTGTCTGTGTGGTCGTGTGTCTGTGTATACCTGTTCAGGTGGGTCAGTGGTTCAGGGCAGGCCCTTAGACGTAGCAGGGCTGGTTGTCATGGAGACGGGGTGCCGCGCAG**GTGCAGGTGCTGGCGGAGAGGGCGGAGGCGGTGCGGACGCGGATCTCGCGCATGGTGGTGGTGATGGTGCTGCTCTTCACCGTCTGCTGGGGCCCCATCCAGCTGTCCGTCCTGTACCAGGCCCTCCACCCGGCCACGCGCAGGAGCTACGCCCTCTACAAACTGAAGATCTGGGCCCACTGCATGTCCTACTCCAGCTCCTCCGTCAACCCCATCATCTACGCCTTCATGGGAGCGAACTTCAGAAAGTCCTTC**AAAAAGGCCTTTCCTTTCATCTTCAAACGCAGGGCCGGGAGAACAGCGGGGGCCTCGGCCAACACAGAGCTGCACTACCTTTCATCCGGAACATGAAGGACCGCAAAAGGCCAGGCTGCATCACACATACACACACACATGTACATGCACACNNNNACACACACACACACACTCCCTCAGGACAGAGAAAACAGGCTAAAAAAAAAAGCAGTGCGTTTGGACACTGACACGCTTATATCCGAC
