# Supplementary material for: Multiple Kisspeptin Receptors in Early Osteichthyans Provide New Insights into the Evolution of This Receptor Family
Source: PLoS One. 2012 Nov 20;7(11):e48931. doi: 10.1371/journal.pone.0048931 (PMC3502363; doi:10.1371/journal.pone.0048931)
Supplement: Figure S2 — Molecular cloning of eel Kissr-3 splicing variants Kissr-3_v2 and Kissr-3_v3. Nucleotide and deduced amino-acid sequence of the cDNAs encoding the eel Kissr-3_v2 (A) and Kissr-3_v3 (B). Nucleotides (top) are numbered from 5′ to 3′. The amino-acid residues (bottom) are numbered beginning with the first methionine residue in the ORF. The asterisk (*) indicates the stop codon. The predicted transmembrane domains (TMD) are underlined. (DOC) [file pone.0048931.s002.doc]

**A European eel Kissr-3_v2**

**1 – ATATTAAAGGCAGGGGACAGACTGGTGCTCCTGCTTTCCACCAGACCGCAACCCCATGGGCTGCTGCCCCACGCTGTGATTGGCTGGGACTCTCAGGCTGCT**

**103 - GCAGGCTCAGTTCTATAGGCCTGGACTTGCCGTGATTGGCTGGCATTGTGAGGACGCCGGTTACCATGGAGGACGGATGGCAACCGAACGGCACCGCCGCTC**

**1 - M E D G W Q P N G T A A P**

**205 - CACCGTGCGACCCGGACGCGGCGTGCAACGGCTCGGCGCTGGCGCTCCTGACTCCGCCCCTGCTGGTGGACGCCTGGCTGGTTCCCCTGTTCTTCGCCATCA**

**14 - P C D P D A A C N G S A L A L L T P P L L V D A W L V P L F F A I I**

**307 - TCATGCTGCTCGGCCTTGTGGGAAACTCCCTGGTCATCTACGTGGTCACCAAGCACCGGCAGATGAAGACGGTCACTAACTTCTACATCGCTGGGTGTTTGG**

**48 - M L L G L V G N S L V I Y V V T K H R Q M K T V T N F Y I A G C L G**

**TMD1**

**409 - GGACTTTATGTGCAGACTGGTCAACTACCTTCAACAGGTGACGGTGCAGGCGACCTGCATCACGCTGTCGGCCATGAGCGTGGATCGCTGCTACGTGACCGT**

**82 - T L C A D W S T T F N R ***

**511 - TTATCCTCTTCAGTCCCTACGCCATCGAACGCCACGCATGGCCATGGCCGTCAGCATCGCCATCTGGACAGGGTCGCTGGGACTCTCTGTGCCTGTGGCGGT**

**613 - GTACCAGCGGCTGGAGACGGGGTACTGGTACGGCCCGCAGGTGTACTGCACCGAGTCCTTCCCCTCCCCCGAGCGCCAGAAGGCCTTCATCCTCTACACCTT**

**715 - CCTGGCGGTCTACCTGCTGCCGCTGCTCACCATCTGCCTGTGCCACGCCTTCATGCTCAAACGCATGGGCCAGCCCGCGGTGGAGCCCGCAGACAACAGCTA**

**817 - CCAGGTGCAGGTGCTGGCGGAGAGGGCGGAGGCGGTGCGGACGCGGATCTCGCGCATGGTGGTGGTGATGGTGCTGCTCTCCACCGTCTGCTGGGGCCCCAT**

**919 - CCAGCTGTCCGTCCTGTACCAGGCCCTCCACCCGGCCACGCGCAGGAGCTACGCCCTCTACAAACTGAAGATCTGGGCCCACTGCATGTCCTACTCCAGCTC**

**1021 - CTCCGTCAACCCCATCATCTACGCCTTCATGGGAGCGAACTTCAGAAAGTCCTTCAGAAAGGCCTTTCCTTTCATCTTCAAACGCAGGGCCGGGAGAACAGC**

**1123 - GGGGGCCTCGGCCAACACAGAGCTGCACTACCTTTCATCCGGAACATGAAGGACCGCAAAAGGCCAGGCTGCATCACACATACACACACACATGTACATGCA**

**1225 - CACACACACACACACACACACACTCCCTCAGGACAGAGAAAACAGGCTAAAAAAAAAAGCAGTGCGTTTGGACACTGACACGCTTATATCCGAC**

**B European eel Kissr-3_v3**

**1 – ATATTAAAGGCAGGGGACAGACTGGTGCTCCTGCTTTCCACCAGACCGCAACCCCATGGGCTGCTGCCCCACGCTGTGATTGGCTGGGACTCTCAGGCTGCT**

**103 - GCAGGCTCAGTTCTATAGGCCTGGACTTGCCGTGATTGGCTGGCATTGTGAGGACGCCGGTTACCATGGAGGACGGATGGCAACCGAACGGCACCGCCGCTC**

**1 - M E D G W Q P N G T A A P**

**205 - CACCGTGCGACCCGGACGCGGCGTGCAACGGCTCGGCGCTGGCGCTCCTGACTCCGCCCCTGCTGGTGGACGCCTGGCTGGTTCCCCTGTTCTTCGCCATCA**

**14 - P C D P D A A C N G S A L A L L T P P L L V D A W L V P L F F A I I**

**307 - TCATGCTGCTCGGCCTTGTGGGAAACTCCCTGGTCATCTACGTGGTCACCAAGCACCGGCAGATGAAGACGGTCACTAACTTCTACATCGGCGACGGTGCAG**

**48 - M L L G L V G N S L V I Y V V T K H R Q M K T V T N F Y I G D G A G**

**TMD1**

**409 - GCGACCTGCATCACGCTGTCGGCCATGAGTGTGGATCGCTGCTACGTGACCGTTTATCCTCTTCAGTCCCTACGCCATCGAACGCCACGCATGGCCATGGCC**

**82 - D L H H A V G H E C G S L L R D R L S S S V P T P S N A T H G H G R**

**511 - GTCAGCATCGCCATCTGGACAGGGTCGCTGGGACTCTCTGTGCCTGTGGCGGTGTACCAGCGGCTGGAGACGGGGTACTGGTACGGCCCGCAGGTGTACTGC**

**116 - Q H R H L D R V A G T L C A C G G V P A A G D G V L V R P A G V L H**

**613 - ACCGAGTCCTTCCCCTCCCCCGAGCGCCAGAAGGCCTTCATCCTCTACACCTTCCTGGCGGTCTACCTGCTGCCGCTGCTCACCATCTGCCTGTGCCACGCC**

**150 - R V L P L P R A P E G L H P L H L P G G L P A A A A H H L P V P R L**

**715 - TTCATGCTCAAACGCATGGGCCAGCCCGCGGTGGAGCCCGCAGACAACAGCTACCAGGTGCAGGTGCTGGCGGAGAGGGCGGAGGCGGTGCGGACGCGGATC**

**184 - H A Q T H G P A R G G A R R Q Q L P G A G A G G E G G G G A D A D L**

**817 - TCGCGCATGGTGGTGGTGATGGTGCTGCTCTCCACCGTCTGCTGGGGCCCCATCCAGCTGTCCGTCCTGTACCAGGCCCTCCACCCGGCCACGCGCAGGAGC**

**218 - A H G G G D G A A L H R L L G P H P A V R P V P G P P P G H A Q E L**

**919 - TACGCCCTCTACAAACTGAAGATCTGGGCCCACTGCATGTCCTACTCCAGCTCCTCCGTCAACCCCATCATCTACGCCTTCATGGGAGCGAACTTCAGAAAG**

**252 - R P L Q T E D L G P L H V L L Q L L R Q P H H L R L H G S E L Q K V**

**1021 - TCCTTCAGAAAGGCCTTTCCTTTCATCTTCAAACGCAGGGCCGGGAGAACAGCGGGGGCCTCGGCCAACACAGAGCTGCACTACCTTTCATCCGGAACATGA**

**286 - L Q K G L S F H L Q T Q G R E N S G G L G Q H R A A L P F I R N M K**

**1123 - AGGACCGCAAAAGGCCAGGCTGCATCACACATACACACACACATGTACATGCACACACACACACACACACACACACTCCCTCAGGACAGAGAAAACAGGCTA**

**320 - D R K R P G C I T H T H T H V H A H T H T H T H T P S G Q R K Q A K**

**1225 - AAAAAAAAAGCAGTGCGTTTGGACACTGACACGCTTATATCCGAC**

**354 - K K S S A F G H ***
