# Supplementary material for: Multiple Kisspeptin Receptors in Early Osteichthyans Provide New Insights into the Evolution of This Receptor Family
Source: PLoS One. 2012 Nov 20;7(11):e48931. doi: 10.1371/journal.pone.0048931 (PMC3502363; doi:10.1371/journal.pone.0048931)
Supplement: Figure S3 — Prediction of four Kissr CDS from the coelacanth genome. Nucleotide and deduced amino-acid sequences of the CDS encoding the coelacanth Kissr-1 (A), Kissr-2 (B), Kissr-3 (C) and Kissr-4 (D). Nucleotides (top) are numbered from 5′ to 3′. The amino-acid residues (bottom) are numbered beginning with the first methionine residue in the ORF. The asterisk (*) indicates the stop codon. The predicted transmembrane domains (TMD) are underlined. The exon-exon junctions are represented by two nucleotides coloured in red. (DOC) [file pone.0048931.s003.doc]

**A Predicted coelacanth Kissr-1**

**1 - ATGTTCGCTGTCTCTGAGAGCCCCCATTTGAATAACTCTTTCCCTTGTTTCAATGATTCTTGCTCAGATGCTGGGCAGTCAGAGTGCACC - 90**

**1 - M F A V S E S P H L N N S F P C F N D S C S D A G Q S E C T - 30**

**91 - AACAGAACTGCTTCCCAAAGCCCCCCATTCCTGGTTGACGCCTGGCTGGTGCCTCTCTTTTTTGCTATCTTGATGGTTGTTGGTCTTGCT - 180**

**31 - N R T A S Q S P P F L V D A W L V P L F F A I L M V V G L A - 60**

**TMD1**

**181 - GGAAACTCTCTAGTCCTATATGTCATTTCCAAACACAAGCAAATGAGGACTGTCACCAACTTCTATATAGCAAATTTGGCTACAACTGAT - 270**

**61 - G N S L V L Y V I S K H K Q M R T V T N F Y I A N L A T T D - 90**

**271 - ATTATTTTCCTAGTATGCTGTGTCCCATTTACAGCAGTGCTGTACCCTCTACCCAGCTGGATCTTTGGAGAGTTCATGTGCAGATTCGTA - 360**

**91 - I I F L V C C V P F T A V L Y P L P S W I F G E F M C R F V - 120**

**TMD2**

**361 - AATTACATCCAGCAGGTATCCATTCAAGCAACTTGTGTCACCCTAACTGCAATGAGTGTAGATCGCTGGTATGTGACAGTGTACCCACTC - 450**

**121 - N Y I Q Q V S I Q A T C V T L T A M S V D R W Y V T V Y P L - 150**

**TMD3**

**451 - CGCTCCCTGAGACAGAGAACACCCCGTGTTGCCGTAGCAGTAAGTCTTGGAATCTGGATAGGCTCCTTTATTGTGTCTATCCCAGTCCCA - 540**

**151 - R S L R Q R T P R V A V A V S L G I W I G S F I V S I P V P - 180**

**TMD4**

**541 - ATGTATCATAAAGTTACAACTGGTTGCTGGTTTGGCCCACAGACCTACTGCAGTGAGTCATTTCCCTCTGTCTCCCATGAGAAGGCACTT - 630**

**181 - M Y H K V T T G C W F G P Q T Y C S E S F P S V S H E K A L - 210**

**631 - ATTCTGTATAATTTCCTGTTGATATACGTACTACCTTTGCTAATAATCTGTGTGTGCTACACAGCTATGCTGTACCATATGAGGCGTCCA - 720**

**211 - I L Y N F L L I Y V L P L L I I C V C Y T A M L Y H M R R P - 240**

**TMD5**

**721 - GCAGTGGAACCCACCGATAATAACTACCAGGTGCAGCTTCTGGCAGAGCGCTCAGAAGCAATGCGCACAAAGATCTCAAGGATGCTGGCA - 810**

**241 - A V E P T D N N Y Q V Q L L A E R S E A M R T K I S R M L A - 270**

**811 - ATGATGGTCTTCCTCTTCGCCATTTGTTGGGGTCCAATTGAACTTTATATTCTATTCCAAGCCTTTAGCCCTAGCTTCCAGAAGAATTAC - 900**

**271 - M M V F L F A I C W G P I E L Y I L F Q A F S P S F Q K N Y - 300**

**TMD6**

**901 - TACACCTACAAAGTAAAGATCTGGGCCCACTGCATGTCCTATACCAATTCTTCTGTCAATCCTATTGTTTATGCATTCATGGGTGCCAAC - 990**

**301 - Y T Y K V K I W A H C M S Y T N S S V N P I V Y A F M G A N - 330**

**TMD7**

**991 - TTCAGAAAGGCCTTCAAGAAAGCTTTTCCATTTATATTCAAGCAGAGGGTTGGGAGCACCAATGTGGCCAATGCCACTGCTAACACTGAG - 1080**

**331 - F R K A F K K A F P F I F K Q R V G S T N V A N A T A N T E - 360**

**1081 - ATGCATTTTGTCTCCTCTGGAACCTAA - 1107**

**361 - M H F V S S G T * - 369**

**B Predicted coelacanth Kissr-2**

**1 - ATGGATTTGTTGAACTTTTCCGCAGACTCTCGAAATCTGTCAGAGCTGAAGGGAAACTTGTCGGAGGTAAACGCGACAGAGGTGGGCTCA - 90**

**1 - M D L L N F S A D S R N L S E L K G N L S E V N A T E V G S - 30**

**91 - CCCCCCTTCCTCACAGATGCCTGGCTGGTGCCCCTTTTCTTTGCGCTCATCATGCTGGTGGGACTCATTGGGAACTCTCTGGTCATCTAC - 180**

**31 - P P F L T D A W L V P L F F A L I M L V G L I G N S L V I Y - 60**

**TMD1**

**181 - GTGATTTCCAAACACAGGCAGCTGAGGACTGCCACCAACTTCTACATAGCTAACTTGGCTACTACTGACATCATCTTCCTGGTGTGCTGT - 270**

**61 - V I S K H R Q L R T A T N F Y I A N L A T T D I I F L V C C - 90**

**TMD2**

**271 - GTCCCCTTCACAGCCACTCTCTACCCTCTACCCAGCTGGATTTTCGGAGAATTCATGTGCAAGTTTGTCGCTTACCTTCAGCAGGTTACA - 360**

**91 - V P F T A T L Y P L P S W I F G E F M C K F V A Y L Q Q V T - 120**

**361 - GTTCAGGCCACGTGCATTACCCTGACAGCAATGAGTGCAGACCGCTGCTATGCCACAGTTTACCCGTTGAAGTCACTGCGCCACCGCACC - 450**

**121 - V Q A T C I T L T A M S A D R C Y A T V Y P L K S L R H R T - 150**

**TMD3**

**451 - CCCAGAGTCGCCATGATTGTCAGTGTCTGCATCTGGATAGGATCCTTTATTTTATCAACACCAATAATCACCTACCAGGAGATAAAGGAA - 540**

**151 - P R V A M I V S V C I W I G S F I L S T P I I T Y Q E I K E - 180**

**TMD4**

**541 - GGTTACTGGTACGGGCCGCGGACTTATTGCATCGAGGAGTTCCCCTCCGACACCTACAAGACAGCATTTATTCTCTATCAGTTTCTGGCT - 630**

**181 - G Y W Y G P R T Y C I E E F P S D T Y K T A F I L Y Q F L A - 210**

**631 - GTCTACCTTCTGCCCCTTCTCACCATTTGTCTCTGTTACTTCTTCATGCTGAAGCGAGTTGGACAGCCCATCGTGAAGCCAGTGGATAAC - 720**

**211 - V Y L L P L L T I C L C Y F F M L K R V G Q P I V K P V D N - 240**

**TMD5**

**721 - AACTATCAGGTTCAGCTATTGTCAGAAAGGACAGTTGCCATGCGAAGCAAGATCTCCAAGATGGTGGTGGTGATTGTCCTGCTCTTCACC - 810**

**241 - N Y Q V Q L L S E R T V A M R S K I S K M V V V I V L L F T - 270**

**811 - ATCTGCTGGGGCCCCATACAGCTTTTCATCCTGTTCCAAGGGTTTTACCCAAAGTTCCAGGTCAATTACGCTACCTACAAGATCAAGACA – 900**

**271 - I C W G P I Q L F I L F Q G F Y P K F Q V N Y A T Y K I K T - 300**

**TMD6**

**901 - TGGGCTAACTGCATGTCCTATGCCAACTCTTCCCTCAACCCCATTGTCTACGGCTTTATGGGTGCCAGCTTCAGGAAGTCCTTCAAGAAG - 990**

**301 - W A N C M S Y A N S S L N P I V Y G F M G A S F R K S F K K - 330**

**TMD7**

**991 - GCATTTCCATTCATGTTCCGGCGCAGGGTGAGGGACAGCAGCATGACCTCTGGAACTGTGAATGCAGAAATGAAATTTGTTGCCACAGAA - 1080**

**331 - A F P F M F R R R V R D S S M T S G T V N A E M K F V A T E - 360**

**1081 - ATGACTCAGAATGAGCTGAAGTGA - 1104**

**361 - M T Q N E L K * - 368**

**C Predicted Coelacanth Kissr-3**

**1 - ATGGCTCTCCAGCACCGCGCCGACCGATCGCCGGGCGCCCCTGCCAATGAGTCCGCCTTCCCCCACCCCGCCTGGGAGGACGCCAAGCAG - 90**

**1 - M A L Q H R A D R S P G A P A N E S A F P H P A W E D A K Q - 30**

**91 - CTGGCCAATCCCGCCCTGGAGGGCTCGGGGGTCCAGCCGACTCTCTTCCTGGCGTCTTACGGCTCGGCTCAGAGTCCCCCTCAGCTGGCG - 180**

**31 - L A N P A L E G S G V Q P T L F L A S Y G S A Q S P P Q L A - 60**

**181 - GATGCCTGGGTAGTGCCTCTTGTCTTCGCCCTGATCATGTTGGTGGGGCTGGTGGGGAACTCTCTGGTCATCTACGTGATCAGCAAACAC - 270**

**61 - D A W V V P L V F A L I M L V G L V G N S L V I Y V I S K H - 90**

**TMD1**

**271 - CGGCGGATGAGGACCGTCACCAACTTCTACATCGCAAACCTGGCCACTACTGACATCATATTCTTGGTATGCTGTGTACCCTTCACTGCC - 360**

**91 - R R M R T V T N F Y I A N L A T T D I I F L V C C V P F T A - 120**

**TMD2**

**361 - GCTCTCTATCCTTTGCCTAGCTGGATTTTTGGAGATTTCATGTGTAGATTTGTCAGCTACCTCCAACAGGTCACAGCTCAGGCAGCTTGC - 450**

**121 - A L Y P L P S W I F G D F M C R F V S Y L Q Q V T A Q A A C - 150**

**TMD3**

**451 - ATTACTCTTACAGCTATGAGTGTTGATCGTTGTTATGCTACAGTGTGGCCCTTGAAGTCTTTGCATCACCGTACCCCTCAAGTGGCTGTG - 540**

**151 - I T L T A M S V D R C Y A T V W P L K S L H H R T P Q V A V - 180**

**541 - GCAGTCAGTCTGAGTATTTGGATAGGCTCTTTTGTATTGTCATTTCCTGTTGCAATGTATCAGAAGCTAGAAAAAGGATATTGGTACGGA - 630**

**181 - A V S L S I W I G S F V L S F P V A M Y Q K L E K G Y W Y G - 210**

**TMD4**

**631 - CCACAGATATATTGCACTGAATCATACCCTTCGGTATATCACAAGAAGGCCTTCATTCTCTATAATTTTCTGGCAGTTTACCTTCTACCA - 720**

**211 - P Q I Y C T E S Y P S V Y H K K A F I L Y N F L A V Y L L P - 240**

**TMD5**

**721 - CTGATTACAATGTGTGCCTGCTATGCTTTCATGCTTAAACGAATGAGTAGGCCCGTCATTGAACCTGCAGACAACAACCATCAGGTACAA - 810**

**241 - L I T M C A C Y A F M L K R M S R P V I E P A D N N H Q V Q - 270**

**811 - CTCCTGGAAAAAATGTCGAAAGCTATGCACACCAAGATTTCTAAGATGGTTGTTGTGATTGTCCTCCTCTTCGCAATCTGTTGGGGACCC - 900**

**271 - L L E K M S K A M H T K I S K M V V V I V L L F A I C W G P - 300**

**TMD6**

**901 - ATTCAGCTGTTTCTCCTGTTTCAAGCTTTTGATGCTAGCTTCAGGAAAAGTTACGAAACATATGCAATCAAAATTTGGGCTCACTGCATG - 990**

**301 - I Q L F L L F Q A F D A S F R K S Y E T Y A I K I W A H C M - 330**

**991 - TCCTACTTCAACTCTTCAATCAACCCAATTGTGTGTGCCTTCATGGGGGCCAATTTCAGAAAGTCCTTCAAAGAGGTGTTTCCATTCAAA - 1080**

**331 - S Y F N S S I N P I V C A F M G A N F R K S F K E V F P F K - 360**

**TMD7**

**1081 - TTCAAGCAACGAGTTGGCAGCACAAGAGATGCTGTTCTTGAAACTCAGGTGTAG - 1131**

**361 - F K Q R V G S T R D A V L E T Q V * - 378**

**D Predicted Coelacanth Kissr-4**

**1 - ATGGTTACATTTGGTACCGAGGCCACGGGTCCCGGTTCAGACAGGGGTAGAGAGCATTGGGGTTTGGGGCCAAATTTGTGGGTCTACAAC - 90**

**1 - M V T F G T E A T G P G S D R G R E H W G L G P N L W V Y N - 30**

**91 - ATGACGGGCGAGGAGACCCCTCCGTTTCTGACCGATGCCTGGCTGGTTCCCCTCTTCTTCGCCCTCATCATGCTGGTGGGGCTCATAGGA - 180**

**31 - M T G E E T P P F L T D A W L V P L F F A L I M L V G L I G - 60**

**TMD1**

**181 - AACTCTCTGGTCATTTATGTCGTCTCTAAGCACCGGCAGATGAGGACAGCCACCAACTTTTACATTGCTAACCTGGCGACTACAGATATT - 270**

**61 - N S L V I Y V V S K H R Q M R T A T N F Y I A N L A T T D I - 90**

**271 - TTATTCCTGGTGTGTTGTGTTCCCTTCACTGCCACCCTCTACCCTTTACCTAGTTGGATTTTTGGAGACTTCATGTGCAAGTTTGTCAAT - 360**

**91 - L F L V C C V P F T A T L Y P L P S W I F G D F M C K F V N - 120**

**TMD2**

**361 - TACCTGCAGCAGGTGACAGTACAGGCCACATGTATTACGCTGACGGTGATGAGTGTGGACAGATGTTATGCTACTCTGTACCCCCTGCAG - 450**

**121 - Y L Q Q V T V Q A T C I T L T V M S V D R C Y A T L Y P L Q - 150**

**TMD3**

**451 - TCGCTCCGACACCGCACCCCCCGAGTGGCTATGGCTGTCAGTCTGGGCGTCTGGATTGGCTCCTTCCTCCTCTCACTGCCCATGGCGATG - 540**

**151 - S L R H R T P R V A M A V S L G V W I G S F L L S L P M A M - 180**

**TMD4**

**541 - TACCACCGGATCGAGGTTGGACTCTGGTATGGGTTACGCACCTACTGTATTGAGGCCTTCCCCACCGAGTCCCAGCAGAAGGGCTTCATC - 630**

**181 - Y H R I E V G L W Y G L R T Y C I E A F P T E S Q Q K G F I - 210**

**631 - CTGTACACCTTCCTGGCCGTCTACCTTTTGCCATTGGTGACTATCTGTGTCTGCTACACTGTCATGCTGAAGAGAGTCGGGAGGCCAGCA - 720**

**211 - L Y T F L A V Y L L P L V T I C V C Y T V M L K R V G R P A - 240**

**TMD5**

**721 - GTGGAACCAGTCAATGACAATTACCAAGTTCAGCACTTATCGGAAAGGTCAGTTGCAATGAGGGCTAAAGTTTCCAGAATGGTGGTGGTG - 810**

**241 - V E P V N D N Y Q V Q H L S E R S V A M R A K V S R M V V V - 270**

**811 - ATGGTCCTCCTTTTCACTGTCTGCTGGGGGCCTATCCAACTCTTCATTCTCTTCCAGGGCTTCTACAAAGACTTTCAGGCCAATTATGAG - 900**

**271 - M V L L F T V C W G P I Q L F I L F Q G F Y K D F Q A N Y E - 300**

**TMD6**

**901 - ACCTATAAGATAAAAACCTGGGCCAACTGCATGTCTTATGCCAACTCAGCCATCAACCCCATTGTCTATGCCTTCATGGGTGATAGCTTC - 990**

**301 - T Y K I K T W A N C M S Y A N S A I N P I V Y A F M G D S F - 330**

**TMD7**

**991 - AGGAAGTCTTTTAAGAAAGCCTTCCCCTTCCTGTTTCAGAGGAGGGTACGAGACAGTTCATTGGCATCAGGGTCCCGAAATGCTGAACTG - 1080**

**331 - R K S F K K A F P F L F Q R R V R D S S L A S G S R N A E L - 360**

**1081 - AAAATTGTTGATGAGGGTACCTGA - 1104**

**361 - K I V D E G T * - 368**
