# Supplementary material for: Multiple Kisspeptin Receptors in Early Osteichthyans Provide New Insights into the Evolution of This Receptor Family
Source: PLoS One. 2012 Nov 20;7(11):e48931. doi: 10.1371/journal.pone.0048931 (PMC3502363; doi:10.1371/journal.pone.0048931)
Supplement: Figure S4 — Prediction of four Kissr CDS from the spotted gar genome. Nucleotide and deduced amino-acid sequences of the CDS encoding the spotted gar Kissr-1 (A), Kissr-2 (B), Kissr-3 (C) and Kissr-4 (D). Nucleotides (top) are numbered from 5′ to 3′. The amino-acid residues (bottom) are numbered beginning with the first methionine residue in the ORF. The asterisk (*) indicates the stop codon. The predicted transmembrane domains (TMD) are underlined. The exon-exon junctions are represented by two nucleotides coloured in red. (DOC) [file pone.0048931.s004.doc]

**A Predicted spotted gar Kissr-1**

**1 - ATGTCCGAGGCGGGCGAGGGCTGCGGGTCTCGGTGCCCCAGTGTCCTGAACGAGTCGTGCGCCTCCGCGCTGCCGTGCTGGAACGGCACG - 90**

**1 - M S E A G E G C G S R C P S V L N E S C A S A L P C W N G T - 30**

**91 - GCCGCCCTGCGCCCGCCGCTCCTGGTGGACGCCTGGCTGGTGCCGCTGTTCTTCGCGCTGCTCATGGCGGTGGGGCTGGCGGGCAACTCT - 180**

**31 - A A L R P P L L V D A W L V P L F F A L L M A V G L A G N S - 60**

**TMD1**

**181 - CTGGTCATCCACGTCATCAGCAAGCACAAGAAGATGAGGACCGCAACAAACTTCTACATAGCTAACCTGGCCACCACGGACATCATTTTC - 270**

**61 - L V I H V I S K H K K M R T A T N F Y I A N L A T T D I I F - 90**

**TMD2**

**271 - CTGGTGTGCTGCGTGCCGTTCACCGCCGCGCTATACCCCCTGCCCAGCTGGGTCTTCGGCGAGTTCATGTGCAAGTTCGTCAGCTACATC - 360**

**91 - L V C C V P F T A A L Y P L P S W V F G E F M C K F V S Y I - 120**

**361 - CAGCAGGTATCGGCTCAGGCTACTTGTGTGACCCTGATGGCACTGGGCGTGGATCGCTGGTACGTCACCGTGTTTCCTCTCAGCTCCCTG - 450**

**121 - Q Q V S A Q A T C V T L M A L G V D R W Y V T V F P L S S L - 150**

**TMD3**

**451 - CGCCGCAGAACGGCGAGGACGGCCGCCACCGTAAGCCTGGGTATCTGGATAGGTTCTTTCCTGGTGTCGGTGCCAGTGCCTGTGTACAGC - 540**

**151 - R R R T A R T A A T V S L G I W I G S F L V S V P V P V Y S - 180**

**TMD4**

**541 - AGCATCACGGAGGGAGAGTGGTATGGACCGCAGGTCTACTGCACTGAAAGCTTCCCCACCNNNNNNNNNNNNNNNNNNNNNNNNNNNNNN - 630**

**181 - S I T E G E W Y G P Q V Y C T E S F P T X X X X X X X X X X - 210**

**TMD5**

**631 - NNNNNNNNNNNNNNNNNNNNNNNNNNNNNNNNNNNNNNNNNNNNNNNNNNNNNNNNNNNNNNNNNNNNNNNNNNNNNNNNNNNNNNNNNN - 720**

**211 - X X X X X X X X X X X X X X X X X X X X X X X X X X X X X X - 240**

**721 - NNNNNNNNNNNNNNNNNNNNNCTGCAGTTCCTGGCGGAGCGCTCAGAGGCCACAAGAACCAAGATCTCGCGCATGGTGGCCGTGATGGTG - 810**

**241 - X X X X X X X L Q F L A E R S E A T R T K I S R M V A V M V - 270**

**811 - CTGCTGTTCACCGTGTGCTGGGGTCCCATCCAGTTCTACATCCTGGTGCAGGCCTTCAGCCCCCAGTTCCAGCACAGCTACTCCACCTAC - 900**

**271 - L L F T V C W G P I Q F Y I L V Q A F S P Q F Q H S Y S T Y - 300**

**TMD6**

**901 - AAGCTGAAGATCTGGGCGCACTGCATGTCCTACGCCAACTCCTGCGTCAACCCCATCGTCTACGCCTTCATGGGCGCCAGCTTCCGCAAG - 990**

**301 - K L K I W A H C M S Y A N S C V N P I V Y A F M G A S F R K - 330**

**TMD7**

**991 - GCCTTCAAGAGGGCGTTCCCCTGCGTCTTCAAGCAGCGTGTGGCTGTGGCCCCAGCTCCACGAGGCAATGCCAATGCCGAGATGCATTTC - 1080**

**331 - A F K R A F P C V F K Q R V A V A P A P R G N A N A E M H F - 360**

**1081 - GTCTCCTCTGGGTCCTAG - 1098**

**361 - V S S G S * - 365**

**B Predicted spotted gar Kissr-2**

**1 - ATGATCAATATCACCGAACTATCAATAAACATCACTGATGTGAACGGTTCTTTGGAGAAGACAGAGGAGGGTTCTCCTCCATTTCTCACA - 90**

**1 - M I N I T E L S I N I T D V N G S L E K T E E G S P P F L T - 30**

**91 - GATGCCTGGCTGGTTCCGTTGTTCTTTTCTCTCATCATGTTGGTGGGACTCGTTGGAAACTCATTAGTCATATACATAATATCCAAACAC - 180**

**31 - D A W L V P L F F S L I M L V G L V G N S L V I Y I I S K H - 60**

**TMD1**

**181 - AGACAGATGCGAACAGCCACCAACTTCTACATAGCAAACCTGGCTACCACTGACATAATATTCCTGGTGTGCTGTGTCCCCTTTACTGCC - 270**

**61 - R Q M R T A T N F Y I A N L A T T D I I F L V C C V P F T A - 90**

**TMD2**

**271 - ACTCTTTACCCTCTGCCGGGCTGGATCTTTGGAGACTTTATGTGCAAATTTGTTGCCTTCCTACAACAGGTTACAGTCCAGGCTACCTGT - 360**

**91 - T L Y P L P G W I F G D F M C K F V A F L Q Q V T V Q A T C - 120**

**TMD3**

**361 - ATCACTCTCACAGCAATGAGCGGTGATCGCTGTTATGCTACAGTATACCCTCTGAAATCCTTACGTCATCGCACACCCCGGGTTGCAATG - 450**

**121 - I T L T A M S G D R C Y A T V Y P L K S L R H R T P R V A M - 150**

**451 - CTTGTTAGCATCTGTATTTGGATTGGGTCCTTTATTCTCTCAACACCTATCATCATGTATCAGAAGATCCAGGAAGGGTACTGGTATGGG - 540**

**151 - L V S I C I W I G S F I L S T P I I M Y Q K I Q E G Y W Y G - 180**

**TMD4**

**541 - CCCAGACTCTACTGCTTAGAAAAGTTCCCATCAAAGATGCATGAGAAAGCCTTCACTCTGTATCAGTTCCTGGCTGTCTACCTCCTGCCT - 630**

**181 - P R L Y C L E K F P S K M H E K A F T L Y Q F L A V Y L L P - 210**

**TMD5**

**631 - CTTCTCACGATTTCCTTATGCTACTACTTCATGCTCAAGAGGGTTGGGAAACCAGTGGTCGAGCCTGTAGACAACAATTACCAGGTCCAG - 720**

**211 - L L T I S L C Y Y F M L K R V G K P V V E P V D N N Y Q V Q - 240**

**721 - GTGCTTTCGGAGAGAACTGTGGCCATGAGAAGCAAAATCTCCAAAATGGTGGTAGTAATCGTCCTGCTTTTCACTATTTGCTGGGGGCCC - 810**

**241 - V L S E R T V A M R S K I S K M V V V I V L L F T I C W G P - 270**

**TMD6**

**811 - ATTCAGCTGTTCATACTATTCCAGTCTTTCTATCCTAACTACAAGGCCAACTACGCCACCTACAAGATTAAGACGTGGGCCAATTGTATG - 900**

**271 - I Q L F I L F Q S F Y P N Y K A N Y A T Y K I K T W A N C M - 300**

**901 - TCTTATGCCAACTCATCCATCAATCCCATTGTCTATGGCTTCATGGGTGTTAGCTTCCGCAAGTCTTTCAAGAAAGCCTTTCCTTTCCTT - 990**

**301 - S Y A N S S I N P I V Y G F M G V S F R K S F K K A F P F L - 330**

**TMD7**

**991 - TTCAGACGTAAGGTGAGGGATGGCAGTGTGACCTCAGGGACAGGGAATGCAGAAATGAAGTTTATTGAAACAGATGTTACCCAAAATGAA - 1080**

**331 - F R R K V R D G S V T S G T G N A E M K F I E T D V T Q N E - 360**

**1081 - GGGAAATGA - 1089**

**361 - G K * - 362**

**C Predicted spotted gar Kissr-3**

**1 - ATGGTGGAAACAACTCCAACTGCAGTGAACTCAGCGAATAATTCTGGACTGTCCTTTTGTCACAATGGCTTGGATCTCAGCATACCCTCA - 90**

**1 - M V E T T P T A V N S A N N S G L S F C H N G L D L S I P S - 30**

**91 - GAAGGATCAGAGGCTGCTTTTTCTCTGGGCTCAGGGTGCAATGGGTCAATTGTCAAAGAGCAACCAAGCCCTCCACTGCTGGTTGATGCC - 180**

**31 - E G S E A A F S L G S G C N G S I V K E Q P S P P L L V D A - 60**

**181 - TGGCTCGTTCCTCTGTTTTTTGCCCTAATTATGTTGGTAGGGCTGATAGGAAACTCTTTAGTGATTTATGTGGTTACTAAGCACCGACAG - 270**

**61 - W L V P L F F A L I M L V G L I G N S L V I Y V V T K H R Q - 90**

**TMD1**

**271 - ATGAGGACTGTCACCAATTTTTACATAGCAAATCTGGCAACAACAGATATCATATTTCTGGTTTGCTGTGTGCCTTTCACTGCTACTCTC - 360**

**91 - M R T V T N F Y I A N L A T T D I I F L V C C V P F T A T L - 120**

**TMD2**

**361 - TACCCACTGCCAAGCTGGGTGTTTGGAGACTTTATGTGCAGAATTGTGAACTATCTGCAACAGGTAACAGCCCAGGCAACATGCATCACC - 450**

**121 - Y P L P S W V F G D F M C R I V N Y L Q Q V T A Q A T C I T - 150**

**TMD3**

**451 - CTAAGTGCGATGAGTGTTGACCGCTGTTATGCCACGGTCTACCCACTGCAATCACTGCGCCACCGCACTCCCAGAGTAGCGATGGCCGTT - 540**

**151 - L S A M S V D R C Y A T V Y P L Q S L R H R T P R V A M A V - 180**

**541 - AGTGTGAGCATCTGGCTAGGTTCTTTTGTTCTGTCTGTGCCAGTTGCCATATACCAGAGGCTGGAGTCAGGATACTGGTATGGACCTCAG - 630**

**181 - S V S I W L G S F V L S V P V A I Y Q R L E S G Y W Y G P Q - 210**

**TMD4**

**631 - ACGTACTGCACAGAATCTTTCCCTTCAGCATACCACCAGAAGGCTTTCATTCTCTATACCTTCCTGGCCGTTTACCTTCTACCCCTTATT – 720**

**211 - T Y C T E S F P S A Y H Q K A F I L Y T F L A V Y L L P L I - 240**

**TMD5**

**721 - ACCATCTGTTTTTGCTATGCATTCATGCTGAAAAGAATGAGCAGGCCAGTTGTTGAACCAGTAGATAACAACTATCAGGTCCAGCTTTTG - 810**

**241 - T I C F C Y A F M L K R M S R P V V E P V D N N Y Q V Q L L - 270**

**811 - GCAGAGAGGTCGGAAGCCATGCGCACAAAGATTTCCAAGATGGTGGTTGTGATCGTCGTGCTCTTTACTATCTGCTGGGGCCCCATTCAG - 900**

**271 - A E R S E A M R T K I S K M V V V I V V L F T I C W G P I Q - 300**

**TMD6**

**901 - TTCTTCATCCTCTTACAGGCCTTCTACTCCAACTTTCGTAGGAGCTATGCGACATACAAGATCAAAATATGGGCTCACTGCATGTCCTAC - 990**

**301 - F F I L L Q A F Y S N F R R S Y A T Y K I K I W A H C M S Y - 330**

**991 - TCCAACTCATCTGTCAATCCGATTGTGTATGCTTTCATGGGGGCCAACTTCAGAAAGTCCTTTAAAAAGGCCTTTCCTTTCATCTTCAAA - 1080**

**331 - S N S S V N P I V Y A F M G A N F R K S F K K A F P F I F K - 360**

**TMD7**

**1081 - CAGCGCGTCGGCACCACTGGAGTTGCAGCTGTGAACACTGAAATGCAATTCGTTTCATCGGGAACATAA - 1149**

**361 - Q R V G T T G V A A V N T E M Q F V S S G T * - 382**

**D Predicted spotted gar Kissr-4**

**1 - ATGGAATTTTTTAACTCCACGCCACCCGCGATCGACGTGGGCTCCGGCAACGACACTGAGCTGTGGACCGCCGCCGGTGCTGAAGTCGCC - 90**

**1 - M E F F N S T P P A I D V G S G N D T E L W T A A G A E V A - 30**

**91 - AGCCGCCGCCCCGTTGGCAGCGAGGACCACCGGTCCCAGCCCGGGGGAAGCCTGCTCGCCGGGGCCGGTGCCGAAGGCGTCGGGACCAGG - 180**

**31 - S R R P V G S E D H R S Q P G G S L L A G A G A E G V G T R - 60**

**181 - CTGTGGATCTACAACGTCACGGGCGAGGAGTCCCCGCCGTTCCTGACCGACGCCTGGCTCGTGCCCTTGTTCTACGCCCTGGTCCTGCTG - 270**

**61 - L W I Y N V T G E E S P P F L T D A W L V P L F Y A L V L L - 90**

**271 - GTGGGGCTCGTGGGAAATTCCCTGGTCATCTACGTCATCAGCAAGCACAGGCAGATGAGGACGGCCACTAACTTCTACATAGCCAACCTG - 360**

**91 - V G L V G N S L V I Y V I S K H R Q M R T A T N F Y I A N L - 120**

**TMD1**

**361 - GCGTGTACAGACATCACCTTCCTGGTGTGCTGCGTGCCCTTCACTGCCACCCTGTACCCCCTGCCCAGCTGGATCTTCGGGGAGTTCATG - 450**

**121 - A C T D I T F L V C C V P F T A T L Y P L P S W I F G E F M - 150**

**TMD2**

**451 - TGCAAGTTTGTCAACTACTTGCAGCAGGTCACTGTGCAGGCCACCTGCATCACCCTGACGGCCATGAGCGTGGACCGTTGCTACGCAACC - 540**

**151 - C K F V N Y L Q Q V T V Q A T C I T L T A M S V D R C Y A T - 180**

**TMD3**

**541 - TTATACCCGCTCCAGTCACTACGGCGCCGTACACCACGTGTTGCCATGGCAGTCAGCGTTGGGATATGGATCGGCTCCCTGCCTCTGTCG - 630**

**181 - L Y P L Q S L R R R T P R V A M A V S V G I W I G S L P L S - 210**

**TMD4**

**631 - CTGCCCATGGCCCTGTACCACCGCATCGAGGTGGGCCTGTGGTACGGCCTGCGCACCTACTGCACGGAGAGGTTCCCCACCGAGGGCCTG - 720**

**211 - L P M A L Y H R I E V G L W Y G L R T Y C T E R F P T E G L - 240**

**721 - CAGAGAGCCTACATCCTCTACACCTTCCTGGCCGCCTACTTGCTGCCCCTCCTCACCATCTGCATCTGCTACACCGTCGTGCTGAAGAGG - 810**

**241 - Q R A Y I L Y T F L A A Y L L P L L T I C I C Y T V V L K R - 270**

**TMD5**

**811 - GTGGCCCGGCCCCTGGTGGAGCCCGCTGACCACAACTACCAGCGGGTGCCAGGGCTGTCGGAGCGCTCGGCGGCGATGCGCGGGCGCGTG - 900**

**271 - V A R P L V E P A D H N Y Q R V P G L S E R S A A M R G R V - 300**

**901 - ACGCGCATGGTGGTGGCCATCGTGCTGCTCTTCACCGTGTGCTGGGGCCCCATCATGCTGTTCATGCTGTGCCAGGGCTTCTACCCCGGG - 990**

**301 - T R M V V A I V L L F T V C W G P I M L F M L C Q G F Y P G - 330**

**TMD6**

**991 - TTCCAGGTGGACTACTACACCTACAAGATCAAGACCTGGGCCAACTGCATGAGCTACGCCAACTCAGCCCTCAACCCCATCGTCTACGCC - 1080**

**331 - F Q V D Y Y T Y K I K T W A N C M S Y A N S A L N P I V Y A - 360**

**TMD7**

**1081 - TTCCTGGGGGAGAGCTTCCGCGCCTCCTTCCGCAAGGCCTTCCCGCTGCTCTTCCGGCGACGGGTGCGGGACGGAG - 1156**

**361 - F L G E S F R A S F R K A F P L L F R R R V R D G - 390**
