# Supplementary figures and images for: Multiple Kisspeptin Receptors in Early Osteichthyans Provide New Insights into the Evolution of This Receptor Family
Source: PLoS One. 2012 Nov 20;7(11):e48931. doi: 10.1371/journal.pone.0048931 (PMC3502363; doi:10.1371/journal.pone.0048931)

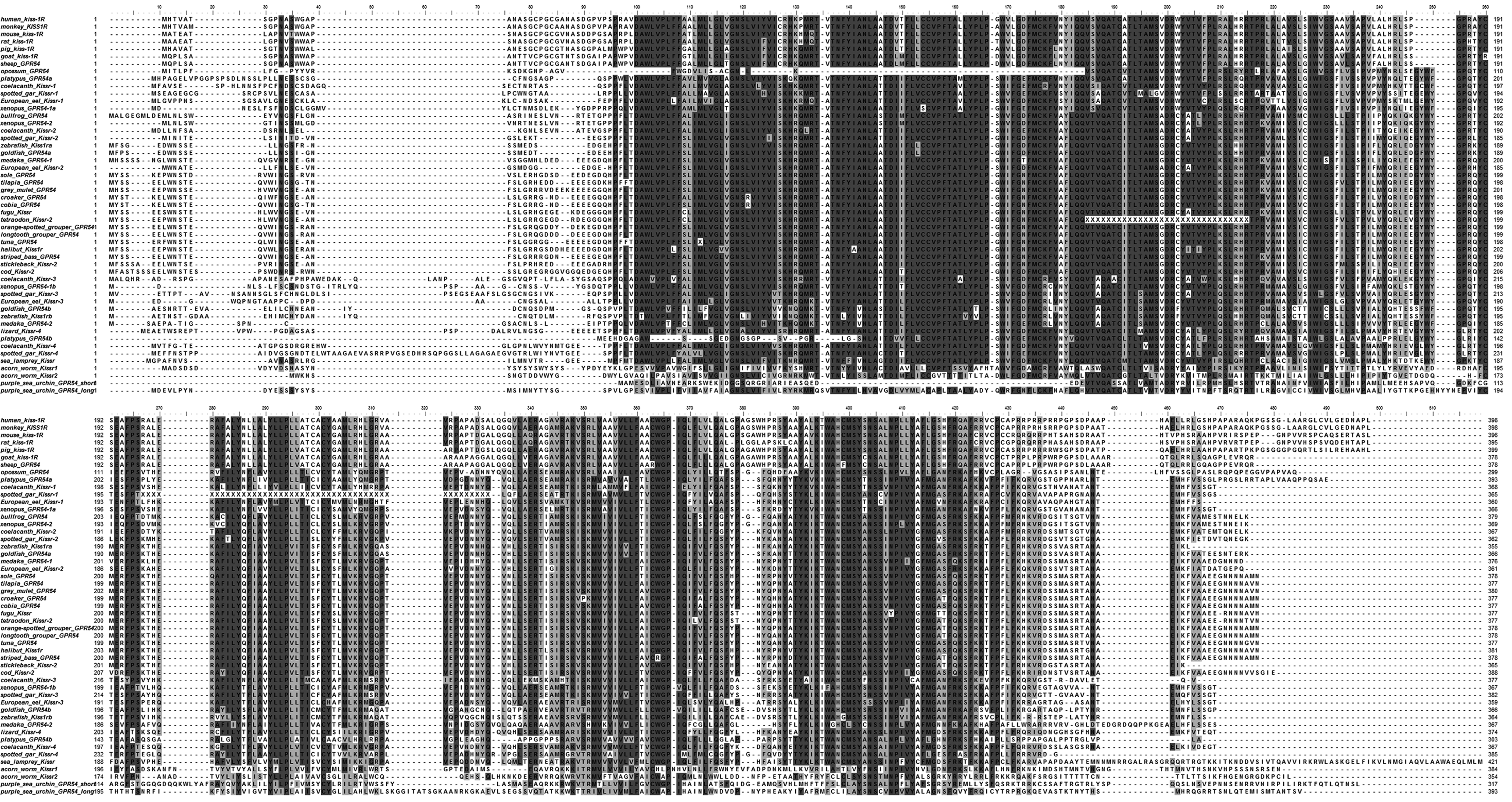

Supplement: Figure S5 — Alignment of the amino-acid sequences of 51 Kissr used for the phylogenetic analysis. The amino-acid sequences were aligned by ClustalW and manually adjusted. The identical amino-acid residues between sequences are shaded in black and the similar (with similar physico-chemical properties) amino-acid residues are shaded in grey. Sequence references are listed in Table S2. (TIF) [file pone.0048931.s005.tif]

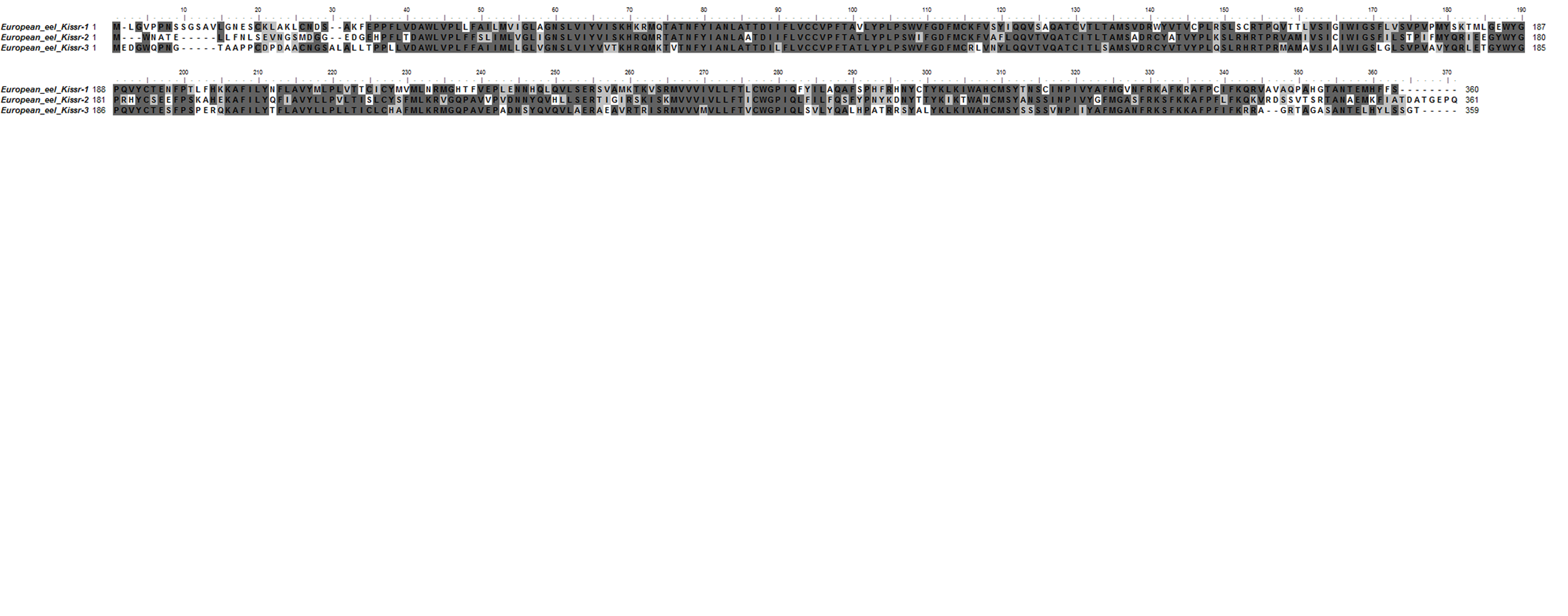

Supplement: Figure S6 — Alignment of the deduced amino-acid sequences of the three eel Kissr. The entire amino-acid sequences were aligned by ClustalW and manually adjusted. The identical amino-acid residues between the three sequences are shaded in black and the similar (with similar physico-chemical properties) amino-acid residues are shaded in grey. (TIF) [file pone.0048931.s006.tif]
